# Associations between Exposures to Perfluoroalkyl Substances and Diabetes, Hyperglycemia, or Insulin Resistance: A Scoping Review

**DOI:** 10.3390/jox11030008

**Published:** 2021-09-14

**Authors:** Rachel Margolis, Karilyn E. Sant

**Affiliations:** School of Public Health, San Diego State University, San Diego, CA 92182, USA; rbmargolis@sdsu.edu

**Keywords:** perfluoroalkyl substances, PFAS, diabetes, hyperglycemia, insulin resistance

## Abstract

Per- and polyfluoroalkyl substances (PFASs) are persistent environmental pollutants that are commonly found in the human body due to exposures via drinking water, surfactants used in consumer materials, and aqueous film-forming foams (AFFFs). PFAS exposure has been linked to adverse health effects such as low infant birth weights, cancer, and endocrine disruption, though increasingly studies have demonstrated that they may perturb metabolic processes and contribute to dysfunction. This scoping review summarizes the chemistry of PFAS exposure and the epidemiologic evidence for associations between exposure to per- and polyfluoroalkyl substances and the development of diabetes, hyperglycemia, and/or insulin resistance. We identified 11 studies on gestational diabetes mellitus, 3 studies on type 1 diabetes, 7 studies on type 2 diabetes, 6 studies on prediabetes or unspecified diabetes, and 15 studies on insulin resistance or glucose tolerance using the SCOPUS and PubMed databases. Approximately 24 reported positive associations, 9 negative associations, 2 non-linear associations, and 2 inverse associations, and 8 reported no associations found between PFAS and all diabetes search terms. Cumulatively, these data indicate the need for further studies to better assess these associations between PFAS exposure and diabetes.

## 1. Introduction

### 1.1. Diabetes: Pathology and Epidemiology

Diabetes is one of the most prevalent diseases worldwide, affecting more than 422 million people globally [1]. Diabetes is a metabolic disease that is characterized by an increase in blood glucose, or hyperglycemia, often due to dysfunctional insulin production or signaling. Insulin is manufactured in pancreatic β cells, carrying out endocrine functions throughout the body. Insulin secretion normally occurs when blood glucose is elevated, signaling the uptake of glucose into cells and tissues. Factors such as physical activity or periods of intermittent fasting that increase cellular glucose demand or decrease bioavailability create a negative feedback loop for insulin secretion via secretion of the counterregulatory hormone glucagon, allowing blood glucose levels to eventually rise again. Ultimately, these coordinated chemosensory processes largely determine the metabolic health of an individual.

Pathologically, there are several types of diabetes, all characterized by hyperglycemia. Insufficient insulin production, often due to β-cell cytotoxicity or death, and resultant hyperglycemia are hallmarks of type 1 diabetes (T1D). When hyperglycemia occurs despite normal insulin production, this is typically characterized as type 2 diabetes (T2D). T2D is often attributed to insulin resistance—a condition commonly characterized by desensitization of insulin receptors, and therefore decreased uptake of glucose into cells [2]. T2D has been largely attributed to lifestyle, including diet and exercise. Another common form of diabetes is gestational diabetes mellitus (GDM), a common endocrine disorder during pregnancy affecting 3–25% of pregnancies [3]. During GDM, pregnant women develop acute insulin resistance, increasing maternal blood glucose levels which then can be transported across the placenta to the fetal tissues. The direct causes of GDM are unknown, though stress hormones and glucagon are frequently elevated, and other common risk factors include age, obesity, and family history of T2D [4,5,6].

Hyperglycemia is easy to detect through simple blood glucose monitoring, including the measurement of fasting blood glucose and glucose monitoring after meals. Diabetes is diagnosed at fasting blood sugar of ≥126 mg/dL. Prediabetes is diagnosed at fasting blood sugar of 100–125 mg/dL [7]. To diagnose diabetes, several models have been developed to relate blood glucose levels to insulin availability and function. Homeostatic model assessment of insulin resistance (HOMA-IR) is a method used to assess insulin resistance. The HOMA-IR test was initially developed in 1985 by Matthews et al. to relate insulin concentrations and glucose levels, and calculated as IR^HOMA^ = I_0_/(22.5 × e^−In(G0)^) [8]. The greater the HOMA-IR, the more insulin resistant someone is. The oral glucose tolerance test (OGTT) is a test for diabetes that is measured twice, once before a sweet drink and once after. Clinical staff will diagnose diabetes by OGTT if blood glucose concentrations ≥200 mg/dL, or prediabetes at 140–199 mg/dL [9]. OGTT is frequently used to detect GDM, diagnosed with an OGTT of 153–199 mg/dL or a fasting plasma glucose of 92–125 mg/dL [10].

Diabetes affects at least 422 million people worldwide. There are 34.2 million cases in the United States, and another 88 million cases of prediabetes (34.5% of the population) [11]. According to the US Centers for Disease Control and Prevention (CDC), the overall diabetes prevalence amongst adults decreased between 2008 and 2018, with an incidence rate of 8.4 per 1000 adults [11]. Comparatively, the CDC discovered an overall increasing trend in diabetes incidence amongst youth. In 2009, T1D affected one in every 518 (1.93 per 1000) youth aged <20 years in the United States, and diabetes overall affected 2.2 per 1000 youths [12]. The SEARCH for Diabetes in Youth (SEARCH) study has highlighted how T2D can be found to occur in all racial/ethnic groups, but the proportion of T1D to T2D can vary greatly by race/ethnicity [13]. Though genetics and lifestyle play large roles in the etiology of diabetes, modeling demonstrates that these causes do not explain the totality of causes of diabetes.

### 1.2. What Are PFAS?

Per-and polyfluoroalkyl substances (PFAS/PFASs) are persistent environmental pollutants commonly found in consumer products, surfactants, and aqueous film-forming foams (AFFFs) [14]. PFAS are fluorinated carbon chains attached to a functional group such as carboxylic acid, sulfonic acid, or sulfonamides, and are primarily non-polar and hydrophobic [15,16,17]. Because of the strength of their C-F bonds, PFAS are highly resistant to processes including hydrolysis, photolysis, biodegradation, and metabolism [18]. In the environment, PFAS have been found in animals in remote locations such as the Artic and Antarctica, showing the vast transport of these compounds through ecosystems [19]. Therefore, the majority of PFAS are widely considered persistent organic pollutants and are a growing environmental and public health threat.

PFAS are often referred to as ‘forever chemicals’ because of their long half-life in environmental and biological matrices. Certain types of PFAS have been found in over 98% of U.S. human blood serum samples [20], and the well-characterized PFAS have biological half-lives in humans ranging from months to years [21,22]. The most common method of exposure to PFAS is through drinking water and increasing PFAS monitoring in surface and drinking water has revealed elevated concentrations globally. Though legacy PFAS such as perfluorooctanesulfonic acid (PFOS) and perfluorooctanoic acid (PFOA) have been phased out of American products for over 20 years, these products are still widely manufactured globally. The lifetime health advisory recommended by the U.S. Environmental Protection Agency is 70 ng/L for PFOA and PFOS combined, but concentrations can be orders of magnitude higher in drinking water near facilities that manufacture fluoropolymers [23]. After phase out, these chemicals are frequently replaced with shorter-chain PFAS alternatives, with little-to-no research available on the potential health and ecological impacts of these emerging compounds [24].

Studies in animals or in vitro models have shed light on the toxicokinetics of PFAS. A plethora of studies have shown the liver to be the primary target organ of PFAS toxicity, though other tissues such as the kidneys and reproductive tract also have pathological consequences of exposures (reviewed in [25]). In animals, exposures to PFAS have also been associated with adverse birth outcomes, including teratogenesis and spontaneous abortion [26,27]. PFOA and PFOS have been associated with several types of cancers, including those of the kidney and reproductive organs, and PFOA is now classified as ‘possibly carcinogenic to humans’ (Class 2B) by the International Agency for Research on Cancer (IARC) [28]. Likewise, PFAS have been detected in cord blood [29,30,31], demonstrating transplacental transfer, and can impact birth weight and predispose offspring to glucose intolerance in adulthood [32]. 

A number of signaling pathways have been shown to be impacted by exposures to PFAS. The most commonly targeted processes include disruption of nuclear receptors including the Constitutive Androstane Receptor (CAR), Peroxisome Proliferator-Activated Receptor α (PPARα), and the Pregnane X Recptor (PXR), as well as pathways governing cell cycle regulation such as p53 signaling (reviewed in [33]). Other studies in vitro have shown PFAS have affinity for both PPARα and PPARγ, Estrogen Receptor α, and the antioxidant response pathway as regulated by Nrf2 [34]. Even with increased affinity for these nuclear receptor pathways, another in vitro study has shown that PFAS exposures to hepatic cells reduce expression of phase I and II detoxification enzymes, including cytochrome P450s (1A2, 2C19, and 3A4), glutathione-S-transferase (M1), and UDP-glucuronosyltransferase (1A1) [35]. Pathologically, both in vivo and in vitro studies have found increased oxidative stress, autophagy, or apoptosis due to PFAS exposures [36,37,38,39]. Despite these many recent advances, many of the health consequences of PFAS exposures are not fully understood, and additional studies are required to comprehensively characterize PFAS toxicity.

### 1.3. PFAS and Diabetic Outcomes in Animal Models

Toxicological studies in animal models have repeatedly shown that PFAS exposures are associated with hyperglycemia and diabetic outcomes. In a 28 day study in mice, PFOA exposure induced insulin sensitivity and glucose tolerance [40], and PFOS exposure increased HOMA-IR [41]. Another murine study found elevated blood glucose without changes in insulin following 28-day exposures to PFOA [42]. Another study found increased insulitis and β-cell apoptosis in non-obese diabetic mice administered perfluoroundecanoic acid (PFUnDA) [43]. Following 7-day exposures in mice, scientists also observed increased oxidative stress and inflammation in islets, further supporting PFOS-induced insulitis [44]. Developmentally, PFOS and perfluorobutanesulfonic acid (PFBS) exposures reduced β-cell area in early pancreatic islets, decreased overall pancreatic size, and decreased insulin gene expression in zebrafish [27,45]. However, these same exposures also led to expanded β-cell area later and increased adiposity in fish later in the lifecourse [46]. Overall, animal studies suggest a link between PFAS exposures, pancreatic damage, and diabetic outcomes.

### 1.4. Scoping Review Goals

There are a growing number of epidemiological studies linking PFAS exposures with adverse metabolic outcomes, including obesity, non-alcoholic fatty liver disease, diabetes, cardiovascular disease, and chronic kidney disease. Because of the strong evidence of an association between PFAS and diabetes in animal models, we will explore the total weight of epidemiological evidence using systematic procedures. In this scoping review, we summarize the current findings from epidemiological and clinical studies on associations between PFAS exposures and the development of diabetes, hyperglycemia, or insulin resistance.

## 2. Materials and Methods

### 2.1. Scoping Review Strategy

A scoping review was performed by searching PubMed and Scopus with the methodology articulated by Arksey and O’Malley [47]. This review was guided by the question, “Does exposure to PFAS increase your chance (risk) of getting a form of diabetes?” The search strategy was developed among the authors, with expertise in toxicology and environmental health, and under consultation with a health science librarian with expertise in systematic review.

### 2.2. Relevant Study Identification

Our search protocol was developed to include all possible articles. Search terms were initially developed on a more general search of the terms PFAS and diabetes. Ultimately, a full list of 20 PFASs were searched after generating a comprehensive list (Table 1). These included perfluorocarboxylic acids (PFCAs), perfluorosulfonic acids (PFSAs), and perfluorosulfonamides. Searches were performed by joining two terms with an AND operator. All PFAS terms were searched separately. The first term was PFAS acronyms linked with an OR to the full text of the word. The second term was diabetes terms linked with OR operators including “diabetes” “insulin resistance” “gestational diabetes” “hyperglycemia” “type 1 diabetes” “type 2 diabetes” OR “prediabetes” (Table 2). Some searches were joined with a third AND operator to search human studies and these searches used “human OR cohort OR adult OR study OR women OR men OR children OR male OR female”.

### 2.3. Study Selection and Charting

**Figure 1:**
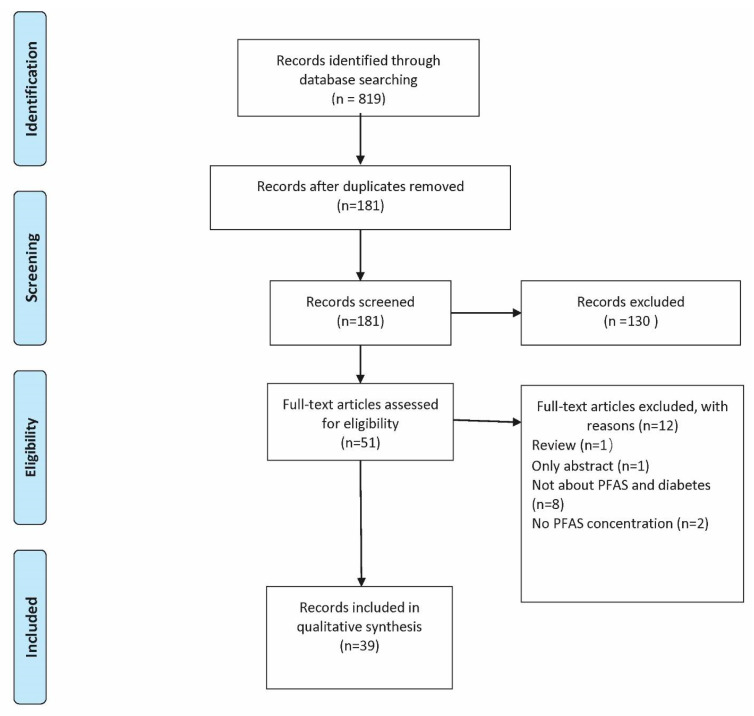
PRISMA flow chart showing search history. PRISMA chart was generated following prescribed systematic and scoping review guidelines (www.prisma-statement.org; accessed on 1 June 2021) [47,48,50].

The references of articles were also screened for potentially relevant studies. From the initial search, 819 articles were found. After removing duplicates, there were 181 articles to screen. After a full-text review, there were 41 articles included. A PRISMA flow chart showing selection and exclusion criteria is shown (Figure 1) [48,49]. From the searches, articles were screened by evaluating titles and abstracts. The full text was reviewed for 51 articles and 45 was included after review. Included studies were required to be (1) human and have a (2) form of diabetes tested for; we required (3) serum, blood, plasma, breast milk, or placental concentrations of PFAS. No limits were placed on publication year or study methodology.

Studies that met the inclusion criteria were manually inputted to a data extraction sheet. The following data were extracted: article title, study population, sample size, PFAS exposure(s), study design, study outcomes and conclusions. The studies were then grouped by the type of diabetes in the study including type 1 diabetes, type 2 diabetes, both type 1 and type 2, no type was stated, hyperglycemia, abnormal fasting blood glucose, or gestational diabetes.

### 2.4. Summation of Results

Title and abstract screening and full-text review required two reviewers for inclusion to the next level and was reviewed by Rachel Margolis for the full-text review. There was a simple yes or no process conducted in EndNote for the title abstract review; difference of opinion for an article was discussed between the two reviewers.

## 3. Results

### 3.1. Gestational Diabetes Mellitus (GDM)

Eleven studies reported results on the relationship between GDM and maternal PFAS concentrations in blood (Table S1). Wang et al. showed that maternal PFAS exposure was not associated with risk of GDM, but it was positively associated with increasing blood glucose levels [51]. Zhang et al. observed a positive association between PFOA and risk for GDM (adjusted odds ratio of 1.86) but reported no association for the other six tested PFASs [41]. Shapiro et al. found no association between PFOA or PFOS and impaired glucose tolerance or GDM. However, they did find a significant positive association between PFHxS and gestational impaired glucose tolerance in the second quartile of exposure with an odds ratio of 3.5 [52]. A study by Matilla-Santander et al. found that PFOS and PFHxS concentrations were positively associated with impaired glucose tolerance with an adjusted odds ratio (per log10 unit increase) of 1.99 and 1.65, respectively, though both PFOS and PFHxS had a non-significant positive association with GDM [53]. Wang et al. found an overall positive association (OR = 1.98) between GDM and PFOA, but not for PFOS [54]. Liu et al. investigated the short-chained perfluorocarboxylates (PFBA, PFPeA, PFHxA, and PFHpA) and observed increased odds for GDM across increasing tertiles (OR: 1.82 and 3.01 in the 2nd and 3rd tertiles compared to the first; *p* = 0.011) [55]. Another study by Xu et al. found a very high adjusted odds ratio for the association between GDM and PFDoA (OR = 13.00; CI: 4.74–24.59), and an odds ratio of 2.02 for PFBS (*p* < 0.01) [56]. Preston et al. also found a positive association between PFOS and glucose levels, but the authors observed a non-monotonic response for PFOSA and glucose levels [57]. 

Of the 11 studies, 3 assessed relationships between PFASs and GDM took metabolic health and family history into consideration in the study designs. Among women with a history of T2D, Rahman et al. found a positive association between GDM and several PFASs, including PFNA, PFOA, PFHpA, and PFDoDA (risk ratios ranged from 1.22 to 3.18) [32]. However, this finding was not significant among women without a family history of T2D. A study by Jensen et al. assessed diabetic phenotypes in women with high risk for GDM, identified as having previously had GDM, having a BMI ≥ 27 kg/m^2^, family history of diabetes mellitus, present multiple pregnancy, glucosuria during pregnancy, or delivery of a macrosomic child [58]. In women classified as high risk for GDM, PFHxS was positively associated with increasing fasting glucose, fasting insulin, and HOMA-IR in a percent change model (ORs = 2.4, 10.9, and 13.5, respectively). PFNA concentration was also associated with increased fasting insulin and HOMA-%β (ORs = 19.1 and 14.7). Another study by Preston et al. showed a moderate association between PFNA and increased odds of an abnormal glucose tolerance test; the results were only significant among overweight and younger women with an odds ratio of 0.76 and 1.48, respectively [57]. Overall, there were 11 studies addressing the link between gestational diabetes and PFAS levels, and most studies found a positive or no association between maternal PFAS concentrations and diabetes risk in pregnancy.

### 3.2. Type 1 Diabetes (T1D)

Three studies have examined the association between serum PFAS concentrations and T1D, with divergent outcomes (Table S2). Conway et al. found negative associations between several PFAS species (PFHxS, PFOA, PFOS, PFNA) and T1D, with adjusted Ors ranging from 0.59 to 0.69 for each PFAS species [59]. Conversely, Predieri et al. found a positive association between serum PFOS concentrations and T1D (*p* < 0.001), with serum PFOS concentrations increased by 178% on average in type 1 diabetics [60]. Steenland et al. found no significant association between serum PFOA concentration and risk of T1D [61]. Each of these studies found a different trend, but also examined different PFAS species in serum. Likewise, these associations were examined in different populations (adults or children), and therefore variables such as age and gender cannot be factored into this scoping review for T1D. The magnitude of effects observed in these studies and their differing outcomes require further elucidation, and more studies are needed to more critically assess this relationship.

### 3.3. Type 2 Diabetes (T2D)

There were overall seven studies that assessed the relationships between PFAS exposures and T2D (Table S3). Of these studies, six measured serum or plasma PFAS concentrations, while Mancini et al. calculated hazard ratios based on estimated mean dietary exposures normalized to body weight [62]. Of the seven studies, three found a significant association between diabetes and PFAS. In the study by Mancini et al., PFOS and PFOA increased T2D hazard ratios in stratified exposure deciles, with the highest hazards being posed to individuals in the central exposure deciles [62]. This suggested an inverse U-shaped response posed by both PFOS and PFOA. Two additional studies showed overall positive associations between plasma PFAS concentrations and T2D. Sun et al. observed that higher plasma concentrations of PFOS and PFOA were associated with higher odds for T2D (ORs = 1.62 and 1.54, respectively), but no significant trends were observed for PFNA, PFDA, and PFHxS [63]. Cardenas et al. also reported a positive association between PFOA and diabetes risk (hazard ratio = 1.14), although the results were not significant for PFOS [64]. Conversely, four studies found no associations between blood PFAS concentrations and T2D [65,66,67,68]. Though both positive and negative associations were observed, studies specifically in cohorts of women found more positive associations [62,63]. 

### 3.4. Prediabetes or Unspecified Diabetes

Six total studies examined the relationship between PFAS serum concentrations and prediabetes or unspecified diabetes (Table S4). Of those six studies, four found positive associations between PFAS and diabetes or prediabetes, while one study found only significant negative associations, and another study found mixed results based on different PFAS species. A cohort study by Conway et al. found negative associations between serum PFHxS and PFOA concentrations and diabetes, but not for serum PFOS and PFNA [59]. A cross-sectional study by Su et al. found that PFOS concentrations were positively associated with impaired glucose homeostasis and the prevalence of diabetes, but PFOA, PFNA, and PFUA each showed a negative association with glucose intolerance and diabetes risk [69]. In a cross-sectional study using data obtained during the National Health and Nutrition Examination Survey (NHANES) study, serum PFOA concentrations were positively associated with diabetes in men, but not women [70]. A cross-sectional study by Lind et al. found that PFNA and PFOA were non-linearly and positively associated with diabetes, with quadratic terms of 1.25 and 1.42 for PFNA and PFOA, respectively [71]. Another cross-sectional study found that serum PFuDA and PFDA, but not PFOS or PFOA, were also associated with increased odds of prediabetes or diabetes [72]. Lastly, a cohort study by Seo et al. found that PFHxS and PFDoDA serum concentrations were positively associated with diabetes prevalence when compared to the non-diabetic levels (*p* < 0.05), though PFNA and PFDA were not significant [17].

### 3.5. Insulin Resistance or Glucose Tolerance

There were a total of 15 studies that examined the relationship between PFAS and insulin resistance or glucose tolerance (Table S5). Of the 15 studies, 7 found a significant association between insulin resistance/glucose tolerance and PFAS. A cross-sectional study by Su et al. found a positive association between PFOS exposure and impaired glucose homeostasis and the prevalence of diabetes, but a negative association was found for PFOA, PFNA and PFUA [69]. A study by Lin et al. found that PFNA was positively associated with hyperglycemia, PFOA was positively associated with increased beta-cell function, and PFOS was positively associated with increased beta-cell function, HOMA-IR, and blood insulin values in the NHANES cohort [16]. A cohort study by Alderete et al. showed an overall positive association between PFOA and PFHxS and 2 h glucose levels with each natural log increase in PFAS concentration [73]. Another study found a positive association between PFOA and PFOS and increased insulin concentration, higher beta-cell activity, and elevated insulin resistance; the results were only significant among overweight children [74]. Liu et al. found a positive association between PFOA (total and linear) and PFOS (branched) and enhanced beta-cell function using data from the NHANES study [75]. A double-blind, randomized, placebo-controlled crossover trial by Kim et al. showed a positive association between PFOS and PFDoDA and insulin resistance; this article showed that vitamin C supplement can help against these effects [30]. Lastly, a study by Cardenas et al. found a small positive association between PFOA and PFOS and HOMA-IR, higher beta-cell function, and higher fasting proinsulin [65].

Three studies found no association between insulin resistance/glucose tolerance and PFAS. A cohort study by Nelson et al. found no association between HOMA-IR and PFOA, PFNA, PFOS, and PFHxS [76]. Two cross-sectional studies found that plasma PFOA, PFOS, PFNA, and PFHxS had no associations with plasma glucose, plasma insulin, or HOMA-IR [77,78]. 

Five studies found a negative association between insulin resistance/glucose tolerance and PFAS. Christensen et al. showed a negative association with PFUnDA and elevated blood glucose levels [24]. A cross-sectional study by Fassler et al. showed that PFOA had an inverse association with HOMA-IR, but the results were not significant [2]. A cohort study by Fleisch et al. showed a negative association between PFOA and insulin resistance levels, but this relationship was more pronounced among females than in males [79]. A study by Koshy et al. found a negative association between PFHxS and insulin resistance [80]. Lastly, a cohort study by Domazet et al. showed that previous childhood exposure at approximately age 9 to PFOS and PFOA is associated with decreased beta-cell function at 15 years of age [81].

## 4. Discussion

The goal of this scoping review was to explore if various PFAS could be linked to the risk of developing diabetes (type 1 and type 2), prediabetes, gestational diabetes, or insulin resistance. The scoping review revealed 39 publications adhering to the inclusion criteria. Approximately 24 reported positive associations, 9 negative associations, 2 non-linear associations, and 2 inverse associations, and 8 reported no associations found out of the 39 included studies. These findings suggest that PFAS and diabetes are most likely to be positively associated.

### 4.1. PFAS and Gestational Diabetes Mellitus (GDM)

We assessed the associations between PFAS exposures and specific diabetic and prediabetic pathologies, including GDM, T1D, T2D, prediabetes, hyperglycemia, and insulin resistance. For gestational diabetes, ten of the eleven included studies showed positive correlations between PFAS and GDM or abnormal glucose tolerance during pregnancy (Table S1). This suggests a direct correlation between serum levels of select PFAS species and risk of developing GDM during pregnancy. 

Beyond the included studies, there is additional evidence that PFAS exposures and GDM are positively associated. Eryasa et al. found that there was a 33% higher PFAS transplacental transfer in mothers with GDM than those without GDM [44]. While our included studies showed a logical relationship between PFAS exposures and GDM incidence, this study suggests that mothers with GDM are also more likely to increase fetal exposures to PFAS. A study by Zong et al. found that lactation helped to reduce maternal PFAS serum concentrations, and that overall lactation was inversely associated with subsequent diabetes risk as a result [82]. While this would effectively reduce maternal PFAS concentrations and potentially risk for diabetes, this relationship demonstrates a concerning source of exposure for developing infants. A monitoring study by Zheng et al. showed that PFAS are routinely detected in breast milk in the United States, demonstrating the scope of this potential concern [83]. Beyond lactation, numerous recent studies have shown that PFAS are frequently detected in cord blood samples, indicating that there is ubiquitous fetal exposure to PFAS globally [84,85,86,87,88,89]. Though the majority of the studies included in this scoping review focused upon adults, more studies are needed to elucidate whether these fetal and infant exposures may increase risk for diabetes in children.

### 4.2. PFAS and Type 1 Diabetes (T1D)

Only three studies specifically assessed the relationship between PFAS and T1D, and these limited studies had conflicting results (Table S2). However, these studies examined different PFAS species and this differences in chemistry may be a potential confounder of this relationship. Additionally, these studies utilized different epidemiological study designs, which can directly impact the conclusions drawn. Conway et al. examined serum PFHxS, PFOA, PFOS, and PFNA with a prospective cohort study design, and found that these PFAS were negatively associated with T1D [59]. Predieri et al. examined only PFOS and PFOA and found a positive association only with PFOS and diabetes in a case-control study [60]. Finally, Steenland et al. used a cross-sectional analysis to find no association between diabetes and PFOA only [61]. PFAS are a very heterogeneous class of compounds, with diverse structural and functional consequences of modifications to the C-F backbone. The length of the C-F chain can also impact the half-life and bioaccumulation factor of PFAS, and therefore the specific species would be expected to play a large role in the toxicokinetics and potentially pathogenesis associated with exposures. While the length of the included compounds only range from C6-C8, the inclusion of perfluorocarboxylic acids (PFCAs) and perfluorosulfonic acids (PFSAs) introduces different chemistries. Ultimately, we are unable to conclude whether PFAS influences risk for T1D based on the current literature.

T1D is an autoimmune disease, in which the immune system destroys insulin-producing β cells of the pancreas (reviewed in [90]). During T1D progression, there is often an initial inflammatory period of β-cell stress, followed by T-cell reactivity and autoimmune destruction of the β cells. There is a growing body of literature demonstrating that PFAS exposures are immunomodulatory, suppressing proinflammatory cytokine production in tissues and decreasing T-cell antibody responses [43,91,92,93,94,95]. Of these studies, Bodin et al. has shown that exposure to PFUnDA increased pancreatic islet inflammation, or insulitis [43]. Therefore, it is possible that the immune system is an important mediator of the association between PFAS and T1D.

### 4.3. PFAS and Type 2 Diabetes (T2D)

Of the seven studies examining the relationship between PFAS and T2D, three found positive associations (Table S3). Overall, these studies mostly completely examined PFOS or PFOA relationships with T2D, but they used several different study designs, including cast-control, cohort, and cross-sectional designs. Overall, we are unable to make a conclusion about the relationship between PFAS exposures and T2D due to variability and limited evidence. However, several studies did investigate comorbidities that may contribute to, or result from, potential interactions between PFAS exposures and diabetes, such as obesity, renal disease, abnormal liver function, and cancers [96,97,98]. Because behavioral factors such as diet and exercise influence development and progression of T2D, it is possible that factors such as consuming convenience foods wrapped in PFAS-containing packaging or spending more sedentary time in contact with surfactant-treated materials could be both increasing PFAS exposures and also increasing risk for diabetes and other comorbidities. Additional studies utilizing cohort designs with more longitudinal monitoring may be able to illuminate the progression of these diseases, timing of comorbidity (before or after T2D), and provide a better temporal characterization of PFAS body burden.

### 4.4. PFAS and Prediabetes, Insulin Resistance, or Glucose Tolerance

Studies assessing the relationship between PFAS exposures and prediabetes, insulin resistance, or glucose tolerance are shown in Tables S4 and S5. These studies primarily replied on clinical measures of hyperglycemia and glucose challenge tests, regardless of diabetes diagnosis. Overall, these studies found modest associations, if any, between PFAS and these outcomes. 

However, one strength of this growing body of literature is the assessment of numerous and diverse PFAS species built into the monitoring and study design. The frequent detection of a myriad of PFAS species highlights the importance of comprehensive PFAS monitoring—especially as longer-chain PFAS are being replaced in industrial and commercial products by shorter chained species. Most studies to date had focused on common PFOA or PFOS monitoring in the United States, and have showed dwindling concentrations in serum since their phase outs [20,99,100,101,102]. However, these phase outs have resulted in increased production of shorter-chain and unsaturated (polyfluorinated) PFAS species, so biomonitoring or non-targeted studies of these emerging PFAS species is necessary. 

### 4.5. Methodological Considerations and Limitations

It is important to consider the study design issues of PFAS and diabetes studies when interpreting the findings of these studies. This review covered numerous cohorts, case-control studies, cross-sectional analyses, and one double-blind randomized clinical trial. Though there were numerous studies using cohort designs, many employed the use of the same project data, including those from the NHANES studies or the C8 Project. Even though these studies used the same data sets and explored the same outcomes, sometimes differing results (significant versus non-significant) were observed based upon analysis methodologies, such as quartile normalization or adjusted regression models. Though standardization of these methods would make studies more comparable for systematic review, often these differences in statistical model help illuminate interesting findings, such as non-monotonic concentration-response relationships, sex-related differences on responses, or diet-toxicant interactions. Likewise, obesity is often a comorbidity along with diabetes, and obesity is also associated with PFAS exposures. While some studies did at least control for obesity as a confounder or moderator of diabetes, there is a need for a greater breadth of research to truly ascertain whether the relationships between PFAS, diabetes, and obesity are strong. One of the obvious limitations observed during this scoping review was the lack of longitudinal data and monitoring studies. Though PFAS have been used in commercial and industrial products for decades, their familiarity to the general public and assessment in scientific studies has been growing exponentially in the past couple of decades. For this reason, the limitation of only having 39 studies that met the inclusion criteria was surprising, and highlights the need for more epidemiological monitoring of PFAS and its potential association with diabetic outcomes.

## 5. Conclusions

In summary, this scoping review found 39 epidemiological studies that assessed the relationship between PFAS and GDM, T1D, T2D, prediabetes, insulin resistance, or glucose tolerance. Of these studies, there is some evidence of a positive association between PFAS and GDM, and variable evidence for other forms of diabetes, insulin resistance, and glucose tolerance. The majority of studies assessed associations between these diabetic outcomes and PFOS or PFOA, legacy PFAS species with prolonged half-lives and bioaccumulative potential in humans. However, there is a need to diversify the PFAS species assessed in biomonitoring studies to represent replacement and/or shorter-chained PFAS species which are becoming increasingly used globally. Likewise, as the number of studies providing evidence for this relationship increases, there is a need for more longitudinal assessments such as prospective cohort studies, and those that employ strategies assessing comorbidities such as obesity. Longitudinal studies could better resolve whether exposures to these ubiquitous PFAS may directly predispose individuals to diabetes.

## Figures and Tables

**Table 1:** Search Terms for PFAS species.

PFAS Search Terms	PFAS Full Text
PFAS	polyfluoroalkyl substances
PFOS	perfluorooctanesulfonic acid
PFOA	perfluorooctanoic acid
PFBA	perfluorobutanoic acid
PFPeA	perfluoropentanoic acid OR perfluorovaleric acid
PFHxA	perfluorohexanoic acid OR perfluorovaleric acid
PFBS	perfluorobutanesulfonic acid
PFNA	perfluorononanoic acid
PFHxS	perfluorohexanesulfonic acid
PFDA	perfluorodecanoic acid
PFUnDA	perfluoroundecanoic acid OR PFUnA
PFDoDA	perfluorododecanoic acid OR perfluorolauric acid
PFTeDA	perfluorotetradecanoic acid
PFPeS	Yielded no results with synonyms
PFHpS	perfluoroheptanesulfonic acid
PFHpA	perfluoroheptanoic acid
FOSAA	perfluorooctane sulfonamidoacetic acid
PFOSA	perfluorooctanesulfonamide

**Table 2:** Search terms for diabetic outcomes.

Outcome (Diabetes)
Diabetes
Gestational diabetes (GDM)
Insulin resistance (IR)
Hyperglycemia
Type 1 diabetes (T1D)
Type 2 diabetes (T2D)
Prediabetes

## Supplementary Materials

The following are available online at https://www.mdpi.com/article/10.3390/jox11030008/s1. Detailed descriptions of search criteria, Table S1: PFAS and Gestational Diabetes Mellitus (GDM); Table S2: PFAS and Type 1 Diabetes; Table S3: PFAS and Type 2 Diabetes; Table S4: PFAS and Prediabetes or Unspecified Diabetes; Table S5: PFAS and Insulin Resistance or Glucose Tolerance.
