# Supplementary material for: Associations between Exposures to Perfluoroalkyl Substances and Diabetes, Hyperglycemia, or Insulin Resistance: A Scoping Review
Source: J Xenobiot. 2021 Sep 14;11(3):115–29. doi: 10.3390/jox11030008 (PMC8482218; doi:10.3390/jox11030008)
Supplement: Supplementary file 1 [file jox-11-00008-s001.zip › jox-1294067-supplementary.pdf]

SUPPLEMENTAL INFORMATION

Associations between Exposures to Perfluoroalkyl Substances and Diabetes, Hyperglycemia, or Insulin Resistance: A Scoping Review

Rachel Margolis and Karilyn E. Sant *

School of Public Health, San Diego State University; San Diego, CA 92182, USA; rbmargolis@sdsu.edu
* Correspondence: ksant@sdsu.edu

TABLE OF CONTENTS

Page	Supplemental Content
PFAS and Diabetes Scoping Review Searches and Criteria	
2	Search terms used for PubMed
2	Search terms used for Scopus
3	Search terms used for Google Scholar
Tables of Studies Included in Scoping Review	
4	Table S1. PFAS & Gestational Diabetes Mellitus (GDM)
15	Table S2: PFAS & Type 1 Diabetes
17	Table S3. PFAS & Type 2 Diabetes
26	Table S4. PFAS & Prediabetes or Unspecified Diabetes
31	Table S5. PFAS & Insulin Resistance or Glucose Tolerance
42	References

PFAS and Diabetes Scoping Review Searches and Criteria

<u>Search terms used for PubMed</u>

Reasoning: The keywords for diabetes represent all possible computations of "diabetes" to yield the max relevant results. The search was conducted separately for each PFAS compound, below are two examples. The compounds were searched separately to yield the most accurate search results and include everything. The complete list of PFAS acronyms and full names searched is shown in Table 1 and Table 2.

(PFHpA OR "Perfluoroheptanoic acid") AND (diabetes OR "gestational diabetes" OR "Insulin Resistance" OR hyperglycemia OR "type 1 diabetes" OR "type 2 diabetes" OR prediabetes)) AND (human OR cohort OR adult OR study OR women OR men OR children OR male* OR female*)

((pfos) OR "Perfluorooctanesulfonic acid") AND (diabetes OR "gestational diabetes" OR "Insulin Resistance" OR hyperglycemia OR "type 1 diabetes" OR "type 2 diabetes" OR prediabetes)) AND (human OR cohort OR adult OR study OR women OR men OR children OR male* OR female*)

<u>Search terms used for Scopus</u>

Reasoning: The same keywords are used as PubMed for the same reasons. The complete list of PFAS acronyms and full names searched is shown in Table 1 and Table 2.

(TITLE-ABS-KEY (diabetes OR "gestational diabetes" OR "Insulin Resistance" OR hyperglycemia OR "type 1 diabetes" OR "type 2 diabetes" OR prediabetes)) AND ((TITLE-ABS-KEY (human OR cohort OR adult OR study) OR TITLE-ABS-KEY (women OR men OR children OR male* OR female*))) AND **(TITLE-ABS-KEY (pfba OR "Perfluorobutanoic acid"))**

(TITLE-ABS-KEY (diabetes OR "gestational diabetes" OR "Insulin Resistance" OR hyperglycemia OR "type 1 diabetes" OR "type 2 diabetes" OR prediabetes)) AND ((TITLE-ABS-KEY (human OR cohort OR adult OR study) OR TITLE-ABS-KEY (women OR men OR children OR male* OR female*))) AND **(TITLE-ABS-KEY (PFNA OR "Perfluorononanoic acid"))**

<u>Search terms used for Google Scholar</u>

Reasoning: Only simple search terms were searched on Google Scholar before the full search strategy was identified. It yielded a very limited number of results and was only used twice.

PFOS and diabetes
hyperglycemia and PFAS

Table S1. PFAS & Gestational Diabetes Mellitus (GDM)

Study Type/ Design<br><br>Study Sample Size (N)	Chemical Exposure(s)	Concentration Ranges and Detection Method(s)	Exposure Assessment	Summary of Results	Measure	Odds Ratio/Data	References
Case-control study<br><br>84 GDM subjects and 168 healthy pregnant women	n-PFOA<br>PFNA<br>PFDA<br>1m-PFOS (perfluoro-1-metylheptylsulfonate)<br>3m + 4m-PFOS (perfluoro-3/4-metylheptylsulfonat)<br>5m-PFOS (perfluoro-5-metylheptylsulfonat)<br>6m-PFOS,<br>n-PFOS<br>PFUnDA<br>PFHXS	Median PFAS concentration with IQR (ng/mL)<br><br><u>GDM</u><br>n-PFOS<br>2.70 (1.87-4.17)<br><br>n-PFOA<br>1.38 (0.98–1.79)<br><br>1m-PFOS<br>0.14 (0.08-0.21)<br><br>3m + 4m-PFOS<br>0.44 (0.25-0.68)<br><br>PFNA<br>0.37 (0.25-0.45)<br><br>PFDA<br>0.26 (0.16-0.44)<br><br>PFUnDA<br>0.26 (0.15-0.43)<br><br>PFHxS<br>0.48 (0.27-0.75)<br><br>5m-PFOS<br>0.36 (0.20-0.60)<br><br>6m-PFOS<br>0.29 (0.21-0.51)<br><br><u>Non-GDM</u><br>n-PFOS<br>2.81 (1.92-4.58)	Maternal serum	Maternal PFAS exposure was not overall associated with risk of GDM. There was a significant positive association between elevated PFAS and increasing blood glucose (hyperglycemia).	OR (95% CI) of the highest category of postpartum fasting blood glucose	1m-PFOS<br>**2.03** (1.09–3.77)<br><br>3m + 4m-PFOS<br>**1.93** (1.04–3.58)<br><br>5m-PFOS<br>**2.48** (1.33–4.65)<br><br>PFHxS<br>**2.26** (1.21–4.21)	[1] Wang, Y., et al., Association of serum levels of perfluoroalkyl substances with gestational diabetes mellitus and postpartum blood glucose. *J Environ Sci (China)*, 2018. 69: p. 5-11.

		n-PFOA<br>1.30 (0.95–1.83)<br><br>1m-PFOS<br>0.14 (0.08-0.24)<br><br>3m + 4m-PFOS<br>0.42 (0.28-0.80)<br><br>PFNA<br>0.36 (0.25-0.52)<br><br>PFDA<br>0.27 (0.19-0.44)<br><br>PFUnDA<br>0.26 (0.16-0.39)<br><br>PFHxS<br>0.47 (0.32-0.74)<br><br>5m-PFOS<br>0.36 (0.21-0.68)<br><br>6m-PFOS<br>0.31 (0.20-0.56)<br><br>Measured by ultra-performance liquid chromatography (UPLC).					
Prospective cohort<br><br>258 eligible pregnant women	PFOA<br>Et-PFOSA-AcOH<br>Me-PFOSA-AcOH<br>PFDeA<br>PFNA<br>PFOSA<br>PFOS	Geometric mean (ng/mL) (95% CI)<br><br>GDM<br>PFOA<br>3.94 (3.15-4.93)<br><br>Et-PFOSA-AcOH<br>0.11 (0.09-0.14)<br><br>Me-PFOSA-AcOH<br>0.30 (0.21-0.42)<br><br>PFDeA	Maternal serum	PFOA was positively associated with an increased risk of GDM.	Adjusted OR (95% CI) for GDM	PFOA<br>**1.86** (1.14–3.02)	[2] Zhang, C., et al., A prospective study of prepregnancy serum concentrations of perfluorochemicals and the risk of gestational diabetes. *Fertil Steril*, 2015. 103(1): p. 184-9.

		0.41 (0.32-0.51)<br><br>PFNA<br>1.23 (0.99-1.52)<br><br>PFOSA<br>0.13 (0.05-0.34)<br><br>PFOS<br>13.10 (10.52-16.33)<br><br><u>Non-GDM</u><br>PFOA<br>3.07 (2.83-3.32)<br><br>Et-PFOSA-AcOH<br>0.11 (0.10-0.12)<br><br>Me-PFOSA-AcOH<br>0.29 (0.26-0.33)<br><br>PFDeA<br>0.40 (0.37-0.43)<br><br>PFNA<br>1.20 (1.12-1.30)<br><br>PFOSA<br>0.11 (0.10-0.12)<br><br>PFOS<br>12.04 (11.12-13.05)<br><br>Measured using isotope dilution high-performance liquid chromatography-tandem mass spectrometry.					
Birth cohort<br><br>1274 pregnant women	PFOS<br>PFHxS<br>PFOA	Geometric Mean (μg/L) (SD)<br><br><u>Gestational IGT</u>	Plasma samples from 1st trimester	PFHxS was associated with an elevated odds ratio for gestational impaired glucose	OR (95% CI) of gestational impaired glucose tolerance in the	PFHxS<br>**3.5** (1.4–8.9)	[3] Shapiro, G.D., et al., Exposure to organophosphorus and organochlorine pesticides,

		PFOS<br>4.58 (1.81)<br><br>PFHxS<br>1.02 (2.31)<br><br>PFOA<br>1.68 (1.80)<br><br>Normal Glucose<br><br>PFOS<br>4.29 (1.60)<br><br>PFHxS<br>1.00 (1.79)<br><br>PFOA<br>1.70 (1.73)<br><br>GDM<br><br>PFOS<br>4.74 (1.67)<br><br>PFHxS<br>1.05 (2.03)<br><br>PFOA<br>1.64 (1.64)<br><br>Measured using UPLC-MS-MS.		tolerance. There was no association found between PFOS and PFOA and IGT or GDM.	second quartile of exposure		perfluoroalkyl substances, and polychlorinated biphenyls in pregnancy and the association with impaired glucose tolerance and gestational diabetes mellitus: The MIREC Study. Environ Res, 2016. 147: p. 71-81.
Cohort<br><br>1540 pregnant women	PFOS<br>PFOA<br>PFHxS<br>PFNA<br>EtFOSAA<br>MeFOSAA<br>perfluorodecanoate<br>Perfluorooctane sulfonamide<br>FOSA	Geometric Mean (ng/L)<br><br>PFOS<br>25.5<br><br>PFOA<br>5.7<br><br>PFHxS<br>2.5<br><br>PFNA<br>1.9	Plasma samples from 1st trimester	PFOS was not associated with glucose tolerance in pregnancy.	OR (95% CI) GDM vs NGT based on comparison of extreme tertiles	PFOS<br>1.5 (0.7-3.0)	[4] Preston, E.V., et al., Associations of Per- and Polyfluoroalkyl Substances (PFAS) With Glucose Tolerance During Pregnancy in Project Viva. J Clin Endocrinol Metab, 2020. 105(8): p. e2864-76.

		MeFOSAA<br>1.9<br><br>EtFOSAA<br>1.2<br><br>Measured using solid phase extraction- HPLC-isotope dilution-tandem mass spectrometry.					
Prospective cohort<br><br>1240 pregnant women	PFOS<br>PFHxS<br>PFOA<br>PFNA	Geometric mean (ng/mL) (SD)<br><br>PFOA<br>2.31 (1.71)<br><br>PFOS<br>5.77 (1.61)<br><br>PFHxS<br>0.55 (1.96)<br><br>PFNA<br>0.64 (1.75)<br><br>Measured by HPLC coupled to tandem mass spectrometry.	Blood plasma samples collected at 13 weeks	PFOS concentrations were positively associated with both impaired glucose tolerance and GDM. PFHxS was positively associated with IGT.	Adj OR (95% CI) (per log10 -unit increase) of impaired glucose tolerance<br><br>Adj OR (95% CI) of GDM	IGT<br>PFOS<br>**1.99** (1.06, 3.78)<br><br>PFHxS<br>1.65 (0.99, 2.76)<br><br>GDM<br>PFOS<br>2.40 (0.93, 6.18)<br><br>PFHxS<br>1.58 (0.73, 3.44)	[5] Matilla-Santander, N., et al., Exposure to Perfluoroalkyl Substances and Metabolic Outcomes in Pregnant Women: Evidence from the Spanish INMA Birth Cohorts. Environ Health Perspect, 2017. 125(11): p. 117004.
Prospective cohort<br><br>560 pregnant women	PFOA<br>PFOS	Median PFAS exposures in the early term of pregnancy (ng/mL) (mean)<br><br>PFOA<br>7.3 (9.6)<br><br>PFOS<br>5.4 (7.8)	Serum samples	PFOA was positively associated with GDM. PFOS was not associated with GDM.	Adj HR (95% CI) of GDM	PFOA<br>1.98 (0.70-5.57)<br><br>PFOS<br>0.71 (0.29-1.75)	[6] Wang, H., et al., Perfluoroalkyl substances, glucose homeostasis, and gestational diabetes mellitus in Chinese pregnant women: A repeat measurement-based prospective study. Environ Int, 2018. 114: p. 12-20.

		Measured by an isotope-dilution method based on ultra-performance liquid chromatography coupled to quadrupole time-of-flight mass spectrometry (UPLC-Q/TOF MS).					
Prospective cohort<br><br>2334 pregnant women between 8-13 weeks of gestation	PFNA<br>PFOA<br>PFHpA<br>PFDoDA<br>NMeFOSAA<br>PFDA<br>PFHxS<br>PFOS	Geometric mean (ng/mL) (95%CI)<br><br><u>Overall Cohort</u><br><br>NMeFOSAA<br>0.09 (0.08-0.09)<br><br>PFDA<br>0.27 (0.26-0.28)<br><br>PFDoDA<br>0.06 (0.06-0.06)<br><br>PFHpA<br>0.08 (0.07-0.08)<br>PFHxS<br>0.76 (0.73-0.78)<br><br>PFNA<br>0.80 (0.78-0.82)<br><br>PFOA<br>1.99 (1.93-2.04)<br><br>PFOS<br>5.21 (5.07-5.35)<br><br>PFUnDA<br>0.20 (0.19-0.20)<br><br><u>GDM</u><br><br>NMeFOSAA<br>0.09 (0.07-0.11)	Plasma samples	PFNA, PFOA, PFHpA and PFDoDA were positively associated with GDM among women with a family history of type 2 diabetes.	RR (range) per 1-SD increment	PFNA<br>**1.43** (1.22, 1.68)<br><br>PFOA<br>**1.27** (1.11, 1.45)<br><br>PFHpA<br>**1.22** (1.12, 1.34)<br><br>PFDoDA<br>**3.18** (1.35, 7.52)	[7] Rahman, M.L., et al., Persistent organic pollutants and gestational diabetes: A multi-center prospective cohort study of healthy US women. Environ Int, 2019. 124: p. 249-258.

		PFDA<br>0.29 (0.24-0.34)<br><br>PFDoDA<br>0.07 (0.06-0.08)<br><br>PFHpA<br>0.09 (0.06-0.12)<br><br>PFHxS<br>0.72 (0.61-0.85)<br><br>PFNA<br>0.85 (0.74-0.98)<br><br>PFOA<br>2.00 (1.74-2.30)<br><br>PFOS<br>5.12 (4.45-5.90)<br><br>PFUnDA<br>0.21 (0.17-0.27)<br><br>Measured by ultra-performance liquid chromatography coupled to electrospray triple quadrupole tandem mass spectrometry.					
Prospective nested case-control study<br><br>439 women	perfluorobutanoate (PFBA)<br>perfluoropentanoate (PFPeA)<br>perfluorohexanoate (PFHxA)<br>perfluoroheptanoate (PFHpA)<br>PFOA, perfluorononanoate (PFNA)<br>perfluorodecanoate (PFDA)<br>perfluoroundecanoate (PFUnDA)	Median (IQR) ng/mL<br><br><u>Non-GDM</u><br><br>1m-PFOS<br>0.12 (0.07-0.22)<br><br>3m-PFOS<br>0.13 (0.08-0.24)<br><br>4m-PFOS<br>0.17 (0.10-0.33)	First trimester maternal serum	There was a positive association found between the sum of the total PFCAs and GDM.	Adj OR (95% CI) of GDM<br><br>Adj OR (95% CI) of GDM, stratified by PFAS tertiles	Short-chained PFCAs (PFBA, PFPeA, PFHxA, PFHpA)<br>**1.99** (1.29, 3.09)<br><br>Total PFCAs<br>**2.47** (1.06-5.74)<br><br><u>By tertile</u><br>Low<br>1.0 (baseline)	[8] Liu, X., et al., Structure-based investigation on the association between perfluoroalkyl acids exposure and both gestational diabetes mellitus and glucose homeostasis in pregnant women. Environ Int, 2019. 127: p. 85-93.

	perfluorododecanoate (PFDoDA)<br>perfluorotridecanoate (PFTrDA)<br>perfluorotetradecanoate (PFTeDA)<br>perfluorobutane atesulfonate (PFBS)<br>perfluoroheptane sulfonate (PFPeS)<br>perfluorohexane sulfonate (PFHxS)<br>perfluorobutane sulfonate (PFHpS)<br>PFOS<br>branched isomers of PFOA (3m-, 4m-, 5m-, 6m-)<br>branched isomers of PFOS (3m-, 4m-, 5m-, 6m-)	5m-PFOS<br>0.32 (0.17-0.58)<br><br>6m-PFOS<br>0.25 (0.17-0.43)<br><br>6m-PFOA<br>0.03 (<LOD-0.05)<br><br>L-PFOS<br>3.05 (2.08-4.54)<br><br>L-PFOA<br>2.18 (1.68- 2.93)<br><br>PFBA<br>0.29 (0.11-0.42)<br><br>PFPeA<br>0.05 (0.03-0.10)<br><br>PFHxA<br>0.01 (<LOD-0.02)<br><br>PFHpA<br>0.01 (<LOD-0.03)<br><br>PFOA<br>2.19 (1.71-2.96)<br><br>PFNA<br>0.43 (0.32-0.57)<br><br>PFDA<br>0.35 (0.25-0.48)<br><br>PFUnDA<br>0.31 (0.21-0.43)<br><br>PFDoDA<br>0.04 (0.01-0.06)<br><br>PFBS<br>0.005 (<LOD-0.006)<br><br>PFHxS				Medium<br>1.82 (0.08-4.16)<br><br>High<br>**3.01** (1.31-6.94)	

		0.28 (0.17-0.45)<br><br>PFOS<br>4.15 (2.71-6.37)<br><br><u>GDM</u><br><br>1m-PFOS<br>0.14 (0.08-0.25)<br><br>3m-PFOS<br>0.15 (0.10-0.25)<br><br>4m-PFOS<br>0.21 (0.12-0.36)<br><br>5m-PFOS<br>0.34 (0.20-0.59)<br><br>6m-PFOS<br>0.28 (0.18-0.45)<br><br>6m-PFOA<br>0.03 (<LOD-0.07)<br><br>L-PFOS<br>3.27 (2.27-4.56)<br><br>L-PFOA<br>2.44 (1.87-3.24)<br><br>PFBA<br>0.35 (0.24-0.41)<br><br>PFPeA<br>0.07 (0.04-0.21)<br><br>PFHxA<br>0.02 (0.02-0.03)<br><br>PFHpA<br>0.02 (<LOD-0.03)<br><br>PFOA<br>2.47 (1.88-3.27)<br><br>PFNA<br>0.50 (0.37-0.59)					

		PFDA<br>0.36 (0.29-0.51)<br><br>PFUnDA<br>0.33 (0.26-0.51)<br><br>PFDoDA<br>0.05 (0.02-0.07)<br><br>PFBS<br>0.005 (<LOD-0.006)<br><br>PFHxS<br>0.30 (0.20-0.46)<br><br>PFOS<br>4.70 (3.01-6.34)<br><br>Measured by ultra-performance liquid chromatography tandem triple-quadrupole mass spectrometry equipped with an HSS PFP column.					
Longitudinal birth cohort<br><br>158 women with high GDM risk and 160 women with low GDM risk matched by gestational age.	PFHxS<br>PFOS<br>PFOA<br>PFNA<br>PFDA	Median (IQR) ng/mL<br><br><u>High GDM Risk</u><br><br>PFHxS<br>0.31 (0.08-0.66)<br><br>PFOS<br>8.37 (3.91-16.50)<br><br>PFOA<br>1.67 (0.67-3.80)<br><br>PFNA<br>0.65 (0.36-1.67)<br><br>PFDA	Serum samples	PFHxS and PFNA were positively associated with increasing fasting glucose/insulin.	Percent change for measures associated with PFHxS and PFNA serum concentrations in women with high risk of GDM, shown through a % change model.	<u>PFHxS</u><br>Fasting glucose<br>2.4 (0.9; 3.9)<br><br>Fasting insulin<br>10.9 (1.7; 20.9)<br><br>HOMA-IR<br>13.5 (3.2; 24.9)<br><br><u>PFNA</u><br>Fasting insulin<br>19.1 (4.9; 35.2)<br><br>HOMA-%β<br>14.7 (3.3; 27.4)	[9] Jensen, R.C., et al., Perfluoroalkyl substances and glycemic status in pregnant Danish women: The Odense Child Cohort. Environ Int, 2018. 116: p. 101-107.

		0.26 (0.15-0.51)<br><br><u>Low GDM Risk</u><br><br>PFHxS<br>0.29 (0.07-0.60)<br><br>PFOS<br>8.15 (4.21-15.53)<br><br>PFOA<br>1.74 (0.71-4.79)<br><br>PFNA<br>0.69 (0.37-1.57)<br><br>PFDA<br>0.27 (0.15-0.62)<br><br>Measured by solid phase extraction followed by LC-MS/MS.					
Nested case-control study<br><br>165 GDM cases and 330 controls (pregnant women)	PFOA<br>PFOS<br>PFBS<br>PFDoA<br>perfluoroundecanoic acid (PFUA)<br>perfluorodecanoic acid (PFDA)<br>perfluoroheptanesulfonic acid (PFHpS)<br>perfluorononanoic acid (PFNA)<br>perfluorohexane sulfonate (PFHxS)<br>perfluorodecanesulfonic acid (PFDS)<br>perfluoroheptanoic acid (PFHpA)<br>perfluorooctane sulfonamide (PFOSA)	Median (IQR) ng/mL<br><br><u>GDM</u><br><br>PFOA<br>8.19 (3.55-13.19)<br><br>PFOS<br>6.69 (3.24-9.42)<br><br>PFBS<br>0.17 (0.09-0.26)<br><br>PFDA<br>1.46 (0.90-2.27)<br><br>PFUA<br>1.13 (0.30-2.21)<br><br>PFNA<br>0.77 (0.20-1.91)	Maternal serum samples	PFBS and PFDoA levels were significantly higher in the GDM group when compared to the controls. PFDoA showed a high odds ratio of 13.00 after modeling for maternal age, sampling time, parity and body mass index [BMI]).	Adj OR (95%) for GDM	PFBS<br>**2.02** (1.04-3.79)<br><br>PFDoA<br>**13.0** (4.74-24.59)	[10] Xu, H., et al., Exposure to elevated per- and polyfluoroalkyl substances in early pregnancy is related to increased risk of gestational diabetes mellitus: A nested case-control study in Shanghai, China. Environment International, 2020. 143: p. 105952.

		PFHpS<br>0.10 (0.08-0.26)<br><br>PFDoA<br>0.19 (0.04-0.33)<br><br>PFHxS<br>1.33 (0.40-1.95)<br><br>PFHpA<br>0.11 (<LOD-0.14)<br><br>PFOSA<br>0.04 (<LOD-<LOD)<br><br>PFDS<br>0.04 (<LOD-<LOD)<br><br>Non-GDM Control<br><br>PFOA<br>7.91 (3.51-12.90)<br><br>PFOS<br>6.45 (3.11-9.20)<br><br>PFBS<br>0.13 (0.07-0.24)<br><br>PFDA<br>1.53 (0.93-2.31)<br><br>PFUA<br>1.09 (0.22-1.93)<br><br>PFNA<br>0.83 (0.21-1.94)<br><br>PFHpS<br>0.10 (0.08-0.25)<br><br>PFDoA<br>0.08 (0.02-0.28)<br><br>PFHxS					

		1.33 (0.41-1.98)<br><br>PFHpA<br>0.07 (<LOD-0.11)<br><br>PFOSA<br>0.04 (<LOD-<br><LOD)<br><br>PFDS<br>0.04 (<LOD-<br><LOD)<br><br>Measured by<br>UPLC-Q/TOF MS.					

Table S2. PFAS & Type 1 Diabetes

Study Type/ Design<br><br>Study Sample Size (N)	Chemical Exposure(s)	Concentration Ranges and Detection Method(s)	Exposure Assessment	Summary of Results	Measure	Odds Ratio/Data	References
Cohort (C8 Health Project)<br><br>6,460 individuals with diabetes and 60,349 without diabetes<br><br>T1D (n=820)<br><br>T2D (n=4,291)<br><br>uncategorized diabetes (n=1,349)	PFHxS<br>PFOA<br>PFOS<br>PFNA	Mean ± SD (ng/mL)<br><br><u>Type 1 diabetes</u><br><br>PFHxS<br>3.4 ± 3.8<br><br>PFOA<br>68.4 ± 176.3<br><br>PFOS<br>21.8 ± 17.1<br><br>PFNA<br>% (n) ng/mL<br>1.4 (0.94)<br><br><u>No diabetes</u><br><br>PFHxS<br>5.2 ± 10.4<br><br>PFOA<br>82.3 ± 227.2<br><br>PFOS<br>23.1 ± 15.4<br><br>PFNA<br>% (n) ng/mL<br>1.6 (0.88)<br><br>Measured by high-performance liquid chromatography/tandem mass spectrometry with triple quadruple mass spectrometry.	Serum samples	PFHxS, PFOA, PFOS and PFNA levels were negatively associated with diabetes in age and sex adjusted analyses.	Adj OR (95% CI) of Type 1 Diabetes	PFHxS<br>**0.59** (0.54–0.64)<br><br>PFOA<br>**0.69** (0.65–0.74)<br><br>PFOS<br>**0.65** (0.61–0.70)<br><br>PFNA<br>**0.65** (0.57–0.74)	[11] Conway, B., K.E. Innes, and D. Long, Perfluoroalkyl substances and beta cell deficient diabetes. *J Diabetes Complications*, 2016. 30(6): p. 993-8.

Case-control study<br><br>25 subjects with type 1 diabetes and 19 healthy controls (children and adolescents)	PFOS<br>PFOA	Median (IQR) (ng/mL)<br><br><u>Type 1 Diabetes</u><br><br>PFOA<br>0.49 (0.48-0.55)<br><br>PFOS<br>0.95 (0.50-1.84)<br><br><u>Controls</u><br><br>PFOA<br>0.48 (0.46-0.50)<br><br>PFOS<br>0.49 (0.48-0.50)<br><br>Measured by high performance liquid chromatography with electrospray ionization (ESI) tandem mass spectrometry.	Serum Samples	PFOS concentrations were significantly higher in type 1 diabetes subjects than in controls.	PFOS concentrations in patients with type 1 diabetes compared to controls<br><br>Mean ± SD	Patients w/ T1D<br><br>PFOS<br>1.53 ± 1.50 ng/mL<br><br>PFOA<br>0.53 ± 0.09 ng/mL<br><br>Controls<br><br>PFOS<br>0.55 ± 0.15 ng/mL<br>$p<0.001$<br><br>PFOA<br>0.50 ± 0.06 ng/mL<br>$p=0.160$	[12] Predieri, B., et al., High Levels of Perfluorooctane Sulfonate in Children at the Onset of Diabetes. International *Journal of Endocrinology*, 2015. 2015: p. 234358.
Cross-sectional analysis<br><br>32,254 U.S. adults	PFOA	Serum PFOA concentration from the C8HP (2005-2006 only), Median (IQR) ng/mL<br><br><u>Community cohort</u><br>24(12-59)<br><br><u>Worker cohort</u><br>113 (56-256)<br><br>Measured by reverse-phase high-performance liquid chromatography/tandem mass spectrometry.	Serum samples	PFOA was found to be not significantly associated with type 1 diabetes.	RR (range) of extreme quartiles of unlagged exposure	PFOA<br>0.54 (0.22, 1.33)<br>p-trend of 0.84	[13] Steenland, K., et al., Ulcerative colitis and perfluorooctanoic acid (PFOA) in a highly exposed population of community residents and workers in the mid-Ohio valley. *Environ Health Perspect*, 2013. 121(8): p. 900-5.

Table S3. PFAS & Type 2 Diabetes

Study Type/ Design<br><br>Study Sample Size (N)	Chemical Exposure(s)	Concentration Ranges and Detection Method(s)	Exposure Assessment	Summary of Results	Measures	Odds Ratio/Data	References
Nested case control study (Nurses' Health Study II)<br><br>116,430 U.S. female nurses	PFOS<br>PFOA<br>PFNA<br>PFHxS<br>PFDA	Median (IQR) ng/mL<br><br><u>Type 2 diabetes</u><br><br>PFOS<br>35.7 (26.4-48.3)<br><br>PFOA<br>4.96 (3.70-6.67)<br><br>PFHxS<br>2.15 (1.35-3.79)<br><br>PFNA<br>0.60 (0.44-0.85)<br><br>PFDA<br>0.13 (0.09-0.19)<br><br><u>Control</u><br><br>PFOS<br>33.1 (23.3-46.8)<br><br>PFOA<br>4.57 (3.35- 6.16)<br><br>PFHxS<br>2.01 (1.32-3.51)<br><br>PFNA<br>0.61 (0.42-0.88)<br><br>PFDA<br>0.16 (0.11-0.23)<br><br>Measured by on-line solid phase	Plasma samples	Higher PFOS and PFOA concentrations were positively associated with an elevated risk of type 2 diabetes. Other PFASs tested were not associated with type 2 diabetes risk.	OR (95% CI) of T2D based on comparison of extreme tertiles	PFOS<br>**1.62** (1.09-2.41)<br><br>PFOA<br>**1.54** (1.04-2.28)<br><br>PFNA<br>0.99 (0.67-1.48)<br><br>PFHxS<br>1.26 (0.86- 1.86)<br><br>PFDA<br>0.71 (0.48-1.05)	[14] Sun, Q., et al., Plasma Concentrations of Perfluoroalkyl Substances and Risk of Type 2 Diabetes: A Prospective Investigation among U.S. Women. *Environ Health Perspect*, 2018. 126(3): p. 037001.

		extraction and liquid chromatography coupled to triple quadropole mass spectrometry.					
Nested Case Control study<br><br>124 pairs	PFOS<br>PFOA<br>PFNA<br>PFHxS<br>PFDA<br>PFUnDA	Median (IQR) ng/mL<br><br><u>Type 2 diabetes</u><br><br>PFOS<br>19.0 (15.0-25.0)<br><br>PFOA<br>2.8 (2.15-3.60)<br><br>PFNA<br>0.55 (0.40-0.76)<br><br>PFHxS<br>0.99 (0.69-1.40)<br><br>PFDA<br>0.21 (<LOQ-0.29)<br><br>PFUnDA<br>0.16 (<LOQ-0.23)<br><br>ΣPFAS<br>24.1 (19.0-31.0)<br><br><u>Controls</u><br><br>PFOS<br>20.0 (16.0-27.0)<br><br>PFOA<br>3.0 (2.3-4.2)<br><br>PFNA<br>0.53 (0.42-0.78)	Plasma samples	There was an overall inverse association between PFAS exposure and risk of type 2 diabetes; however, most of the data observed was non-significant.	OR (95% CI) of T2D based on comparison of extreme tertiles for sum of PFAS (the Σ z-score)	Sum of PFAS<br>0.52 (0.20-1.36)	[15] Donat-Vargas, C., et al., Perfluoroalkyl substances and risk of type II diabetes: A prospective nested case-control study. *Environ Int*, 2019. 123: p. 390-398.

		PFHxS<br>1.10 (0.76-1.40)<br><br>PFDA<br>0.23 (0.17-0.30)<br><br>PFUnDA<br>0.18 (<LOQ-0.26)<br><br>ΣPFAS<br>26.8 (20.3-34.2)<br><br>Measured by liquid chromatography-triple quadrupole mass spectrometry (LC-MS/MS).					
Unique cohort study<br><br>4,129 individuals	PFOA	Deciles of estimated PFOA concentration (min-max) (μg/mL·year)<br><br><u>Type 2 diabetes</u><br><br>PFOA<br><br>Decile 1<br><0.105<br><br>Decile 2<br>0.105-0.146<br><br>Decile 3<br>0.147-0.177<br><br>Decile 4<br>0.178-0.242<br><br>Decile 5<br>0.243-0.363<br><br>Decile 6<br>0.364-0.619	Serum samples	There was no association observed between PFOA exposure and the incidence of type 2 diabetes.	HR (95% CI) of T2D incidence comparing deciles to the lowest decile of PFOA serum concentrations. (D1 denotes first decile [1-10th percentile], etc.)	D1-D2<br>0.91 (0.76–1.08)<br><br>D1-D3<br>1.18 (0.99–1.40)<br><br>D1-D4<br>0.96 (0.81–1.15)<br><br>D1-D5<br>1.04 (0.87–1.24)<br><br>D1-D6<br>1.11 (0.93–1.32)<br><br>D1-D7<br>1.06 (0.89–1.26)<br><br>D1-D8<br>1.00 (0.85–1.19)<br><br>D1-D9<br>1.03 (0.86–1.23)<br><br>D1-D10<br>1.01 (0.84–1.20)	[16] Karnes, C., A. Winquist, and K. Steenland, Incidence of type II diabetes in a cohort with substantial exposure to perfluorooctanoic acid. *Environ Res*, 2014. 128: p. 78-83.

		Decile 7<br>0.620-1.406<br><br>Decile 8<br>1.407-3.79<br><br>Decile 9<br>3.8-7.73<br><br>Decile 10<br>>7,73<br><br>Estimated exposures of PFOA based on self-reported location history, drinking water intake, DuPont emission patterns, and other environmental factors.					
Prospective cohort study<br><br>2,680 women	PFOS<br>PFOA	Deciles (min-max) (ng/kg body weight/day)<br><br><u>PFOA</u><br><br>D1<br>0.08-0.45<br><br>D2<br>0.46-0.55<br><br>D3<br>0.56-0.64<br><br>D4<br>0.65-0.72<br><br>D5<br>0.73-0.80<br><br>D6<br>0.81-0.89	Mean dietary exposure to PFAS (ng/kg body weight/day)	PFOS dietary exposure was nonlinearly associated to type 2 diabetes for non-obese women only. PFOA dietary exposure and type 2 diabetes was characterized by an inverse U-shaped association.	Adj HR (95% CI) of T2D incidence among women comparing deciles to the lowest decile of PFOA serum concentrations. (D1 denotes first decile [1-10th percentile], etc.). Adjusted for BMI.	<u>PFOA</u><br>D1-D4<br>**1.21** (1.06–1.46)<br><br>D1-D5<br>**1.35** (1.15–1.59)<br><br>D1-D6<br>**1.33** (1.05–1.41)<br><br><u>PFOS</u><br>D1-D6<br>**1.46** (1.09–1.96)<br><br>D1-D8<br>**1.52** (1.09–2.11)<br><br>D1-D10<br>**1.44** (1.01–2.06)	[17] Mancini, F.R., et al., Nonlinear associations between dietary exposures to perfluorooctanoic acid (PFOA) or perfluorooctane sulfonate (PFOS) and type 2 diabetes risk in women: Findings from the E3N cohort study. *Int J Hyg Environ Health*, 2018. 221(7): p. 1054-1060.

		D7<br>0.90-1.00<br><br>D8<br>1.01-1.13<br><br>D9<br>1.14-1.34<br><br>D10<br>1.65-3.96<br><br><u>PFOS</u><br><br>D1<br>0.05-0.29<br><br>D2<br>0.30-0.34<br><br>D3<br>0.35-0.39<br><br>D4<br>0.40-0.43<br><br>D5<br>0.44-0.47<br><br>D6<br>0.48-0.51<br><br>D7<br>0.52-0.56<br><br>D8<br>0.57-0.62<br><br>D9<br>0.63-0.72<br><br>D10<br>0.73-1.89<br><br>Measured the mean dietary exposure to PFAS					

		by multiplying the quantities consumed of each food by the contamination level and then dividing by the body weight of each participant.					
Cohort (C8 Health Project)<br><br>6,460 individuals with diabetes and 60,349 without diabetes<br><br>T1D (n=820)<br><br>T2D (n=4,291)<br><br>uncategorized diabetes (n=1,349)	PFHxS<br>PFOA<br>PFOS<br>PFNA	Mean ± SD (ng/mL)<br><br><u>Type 2 diabetes</u><br><br>PFHxS<br>3.8 ± 4.6<br><br>PFOA<br>92.8 ± 400.7<br><br>PFOS<br>25.2 ± 17.0<br><br>PFNA<br>% (n) ng/mL<br>1.5 (0.82)<br><br><u>No diabetes</u><br><br>PFHxS<br>5.2 ± 10.4<br><br>PFOA<br>82.3 ± 227.2<br><br>PFOS<br>23.1 ± 15.4<br><br>PFNA<br>% (n) ng/mL<br>1.6 (0.88)<br><br>Measured by high-performance liquid	Serum samples	PFHxS, PFOA, PFOS and PFNA levels were negatively associated with diabetes in age and sex adjusted analyses.	Adj OR (95% CI)<br>Type 2 Diabetes	PFHxS<br>**0.74** (0.71–0.77)<br><br>PFOA<br>**0.87** (0.89–0.91)<br><br>PFOS<br>**0.86** (0.82–0.90)<br><br>PFNA<br>0.94 (0.88–1.00)	[11] Conway, B., K.E. Innes, and D. Long, Perfluoroalkyl substances and beta cell deficient diabetes. *J Diabetes Complications*, 2016. 30(6): p. 993-8.

		chromatography/tandem mass spectrometry with triple quadruple mass spectrometry.					
Cross-sectional analysis<br><br>1,055 individuals	PFOA	Median of validated type 2 diabetes (ng/mL)<br><br><u>PFOA</u><br><br>32.6<br><br>Deciles of PFOA (min-max) (ng/mL)<br><br><u>PFOA</u><br><br>D1<br><7.9<br><br>D2<br>8.0-11.6<br><br>D3<br>11.7-15.6<br><br>D4<br>15.7-20.8<br><br>D5<br>20.9-28.0<br><br>D6<br>28.1-39.4<br><br>D7<br>39.5-57.5<br><br>D8<br>57.6-89.7<br><br>D9<br>89.8-191.2	Serum samples	The authors found no association between PFOA and either type 2 diabetes or fasting glucose level.	OR (95% CI) for T2D, stratified by PFOA decile.	D1-D2<br>1.00<br><br>D1-D3<br>0.71 (0.48, 1.05)<br><br>D1-D4<br>**0.60** (0.40, 0.89)<br><br>D1-D5<br>**0.65** (0.45, 0.94)<br><br>D1-D6<br>**0.65** (0.46, 0.93)<br><br>D1-D7<br>0.87 (0.62, 1.22)<br><br>D1-D8<br>**0.58** (0.41, 0.82)<br><br>D1-D9<br>**0.62** (0.44, 0.86)<br><br>D1-D10<br>0.72 (0.52, 1.00)	[18] MacNeil, J., et al., A cross-sectional analysis of type II diabetes in a community with exposure to perfluorooctanoic acid (PFOA). *Environ Res*, 2009. 109(8): p. 997-1003.

		D10<br>>191.2<br><br>Measured by HPLC coupled to triple quadrupole mass spectrometry.					
Cross-sectional analysis (Diabetes Prevention Program)<br><br>957 individuals	Branched and linear isomers of PFOS and PFOA; PFOA<br>n-PFOS<br>Sm-PFOS<br>Sm2-PFOS<br>PFOA<br>n-PFOA<br>Sb-PFOA<br>PFHxS<br>EtFOSAA<br>MeFOSAA<br>PFNA	Geometric mean (IQR) ng/mL<br>Lifestyle<br><br>Sum PFAS<br>39.59 (30.10)<br><br>PFOS<br>27.17 (23.50)<br><br>n-PFOS<br>19.13 (17.10)<br><br>Sm-PFOS<br>7.43 (6.40)<br><br>Sm2-PFOS<br>0.13 (0.23)<br><br>PFOA<br>4.90 (3.12)<br><br>n-PFOA<br>4.37 (2.80)<br><br>Sb-PFOA<br>0.44 (0.60)<br><br>PFHxS<br>2.51 (2.70)<br><br>EtFOSAA<br>1.14 (1.50)<br><br>MeFOSAA<br>0.95 (1.20)<br><br>PFNA<br>0.54 (0.40)	Plasma samples	Doubling in PFOA concentration was associated with an increase in diabetes risk and diet may play a role in helping these effects.	HR (95% CI) of T2D based on a doubling of PFOA or PFOS concentration	PFOA<br>1.14 (1.04-1.25)<br><br>PFOS<br>0.92 (0.80- 1.07)	[19] Cardenas, A., et al., Associations of Perfluoroalkyl and Polyfluoroalkyl Substances With Incident Diabetes and Microvascular Disease. *Diabetes Care*, 2019. 42(9): p. 1824-1832.

		Placebo<br><br>Sum PFAS<br>37.30 (27.40)<br><br>PFOS<br>25.60 (20.90)<br><br>n-PFOS<br>17.74 (15.75)<br><br>Sm-PFOS<br>7.21 (6.60)<br><br>Sm2-PFOS<br>0.14 (0.23)<br><br>PFOA<br>4.74 (3.12)<br><br>n-PFOA<br>4.21 (2.80)<br><br>Sb-PFOA<br>0.44 (0.50)<br><br>PFHxS<br>2.30 (2.0)<br><br>EtFOSAA<br>1.11 (1.40)<br><br>MeFOSAA<br>0.93 (1.20)<br><br>PFNA<br>0.52 (0.40)<br><br>Measured by solid-phase extraction–high-performance liquid chromatography–isotope dilution–tandem mass spectrometry.					

Table S4. PFAS & Prediabetes or Unspecified Diabetes

Study Type/ Design<br><br>Study Sample Size (N)	Chemical Exposure(s)	Concentration Ranges and Detection Method(s)	Exposure Assessment	Summary of Results	Measures	Odds Ratio/Data	References
Cross-sectional analysis (NHANES)<br><br>7,904 men	PFOA<br>PFOS<br>PFNA<br>PFHxS	Mean ± SE (ng/mL)<br><br><u>Female</u><br><br>PFOA<br>3.46 ± 0.04<br><br>PFOS<br>14.51 ± 0.26<br><br>PFHxS<br>1.94 ± 0.04<br><br>PFNA<br>1.30 ± 0.03<br><br><u>Male</u><br><br>PFOA<br>4.50 ± 0.06<br><br>PFOS<br>20.80 ± 0.32<br><br>PFHxS<br>2.88 ± 0.05<br><br>PFNA<br>1.52 ±0.02<br><br>Measured by online solid-phase extraction–high performance liquid chromatography–turbo ion spray-tandem mass spectrometry (SPE-HPLC-TIS-MS/MS.	Serum samples	Serum PFOA was positively associated with diabetes in men, but not women.	OR (95% CI) for T2D in men based on comparison of quartile 4 and 1	PFOA<br>**2.67** (1.63–4.38)	[20] He, X., et al., PFOA is associated with diabetes and metabolic alteration in US men: National Health and Nutrition Examination Survey 2003-2012. *Sci Total Environ*, 2018. 625: p. 566-574.

Cross-sectional analysis<br><br>1,016 men and women aged 70 years	PFHpA<br>PFHxS<br>L-PFOS<br>PFOA<br>PFNA<br>PFOSA<br>PFUnDA	Median (IQR) (ng/mL)<br><br>PFHpA<br>0.05 (0.03-0.09)<br><br>PFHxS<br>2.1 (1.6-3.4)<br><br>L-PFOS<br>13.2 (10.0-17.8)<br><br>PFOA<br>3.3 (2.5-4.4)<br><br>PFNA<br>0.7 (0.5-1.0)<br><br>PFOSA<br>0.11 (0.07-0.17)<br><br>PFUnDA<br>0.3 (0.2-0.4)<br><br>Measured by UPLC-MS/MS.	Serum samples	PFNA and PFOA were nonlinearly associated with diabetes.	OR (95% CI) for diabetes, adjusted for multiple confounders	<u>PFNA</u><br>Linear Term<br>1.96 (1.19, 3.22)<br><br>Quadratic Term<br>1.25 (1.08, 1.44)<br><br><u>PFOA</u><br>Linear Term<br>0.62 (0.37, 1.07)<br><br>Quadratic Term<br>1.42 (1.08, 1.86)	[21] Lind, L., et al., Circulating levels of perfluoroalkyl substances and prevalent diabetes in the elderly. *Diabetologia*, 2014. 57(3): p. 473-9.
Cross-sectional analysis<br><br>571 adults	PFOA<br>PFOS<br>PFNA<br>PFUA	Median (IQR) (ng/mL)<br><br><u>Non-diabetes</u><br><br>PFOA<br>8.1 (6.0-10.6)<br><br>PFOS<br>3.1 (2.3-4.4)<br><br>PFNA<br>3.8 (2.7-5.1)<br><br>PFUA<br>6.5 (3.9-9.3)<br><br><u>Diabetes</u><br><br>PFOA<br>5.4 (2.5-7.6)	Serum samples	PFOS exposure was positively associated with impaired glucose homeostasis and the prevalence of diabetes.<br><br>PFOA, PFNA, and PFUA showed a negative association with glucose intolerance and diabetes risk.	Adjusted OR (95% CI) for diabetes when comparing extreme quartiles	PFOS<br>**3.37** (1.18–9.65)<br><br>PFOA<br>**0.16** (0.05–0.50)<br><br>PFNA<br>**0.31** (0.11–0.88)<br><br>PFUA<br>**0.23** (0.08–0.64)	[22] Su, T.C., et al., Serum perfluorinated chemicals, glucose homeostasis and the risk of diabetes in working-aged Taiwanese adults. *Environ Int*, 2016. 88: p. 15-22.

		PFOS<br>5.2 (3.6-8.1)<br><br>PFNA<br>3.1 (1.2-4.8)<br><br>PFUA<br>4.3 (2.1-7.2)<br><br>Measured by UPLC coupled to triple quadrupole mass spectrometry.					
Cohort study<br><br>786 adults with diabetes (n = 44)	PFBS<br>PFHxA<br>PFHpA<br>PFHxS<br>PFOA<br>PFNA<br>PFOS<br>PFDA<br>PFUnDA<br>PFDS<br>PFDoDA<br>PFTrDA<br>PFTeDA	Median (Range) (ng/mL)<br><br><u>All samples</u><br><br>PFBS<br>0.00 (<LOD-0.45)<br><br>PFHxA<br>0.03 (<LOD-3.59)<br><br>PFHpA<br>0.08 (<LOD-1.11)<br><br>PFHxS<br>7.11 (0.51-48.43)<br><br>PFOA<br>4.06 (0.39-37.79)<br><br>PFNA<br>1.74 (<LOD-12.64)<br><br>PFOS<br>10.51 (0.69-70.09)<br><br>PFDA<br>1.13 (<LOD-5.36)<br><br>PFUnDA<br>1.54 (<LOD-9.80)<br><br>PFDS	Serum samples	PFHxS and PFDoDA increased concentrations were positively associated with diabetes prevalence when compared to the nondiabetic levels. PFOS and PFOA were not significant.	Diabetes prevalence and PFAS concentrations when compared to nondiabetic levels	PFHxS<br>PFDoDA<br>P <0.05	[23] Seo, S.-H., et al., Influence of exposure to perfluoroalkyl substances (PFASs) on the Korean general population: 10-year trend and health effects. *Environment International*, 2018. 113: p. 149-161.

		0.04 (<LOD-0.29)<br><br>PFDoDA<br>0.27 (<LOD-2.87)<br><br>PFTrDA<br>0.53 (<LOD-3.41)<br><br>PFTeDA<br>0.04 (<LOD-7.69)<br><br>Measured by HPLC coupled to triple quadrupole mass spectrometry.					
Cross-sectional study<br><br>111 adult men	PFDA<br>PFHpS<br>PFHxS<br>PFNA<br>PFOA<br>PFOS<br>PFuDA	Median (IQR) (ng/mL)<br><br><u>Study Population</u><br><br>PFDA<br>0.52 (0.34-0.90)<br><br>PFHpS<br>0.49 (0.33-0.75)<br>PFHxS<br>1.80 (1.10-2.80)<br><br>PFNA<br>1.40 (0.89-2.30)<br><br>PFOA<br>2.50 (1.80-3.50)<br><br>PFOS<br>19.00 (9.80-28.00)<br><br>PFuDA<br>0.29 (0.17-0.50)<br><br><u>NHANES</u><br><br>PFDA<br>0.23 (0.18-0.28)<br><br>PFHpS<br>Not measured	Serum samples	PFuDA, PFNA and PFDA were all associated with increased risk of pre-diabetes and/or diabetes.	OR (95% CI) for pre-diabetes or diabetes	PFuDA<br>5.29(1.55,18.67)<br><br>PFNA<br>1.37(0.78,1.6)<br><br>PFDA<br>1.91(1.02,3.7)	[25] Christensen, K.Y., et al., Perfluoroalkyl substances in older male anglers in Wisconsin. Environment International, 2016. 91: p. 312-318.

		PFHxS<br>1.66 (1.26-2.05)<br><br>PFNA<br>0.93 (0.72-1.15)<br><br>PFOA<br>2.62 (2.41-2.82)<br><br>PFOS<br>10.33 (8.86-11.80)<br><br>PfuDA<br>0.13 (0.08-0.18)<br><br>Measured by HPLC coupled to triple quadrupole mass spectrometry.					
Cohort (C8 Health Project)<br><br>6,460 individuals with diabetes and 60,349 without diabetes<br><br>T1D (n=820)<br><br>T2D (n=4,291)<br><br>uncategorized diabetes (n=1,349)	PFHxS<br>PFOA<br>PFOS<br>PFNA	Mean ± SD (ng/mL)<br><br><u>Uncategorized diabetes</u><br>PFHxS<br>4.2 ± 4.9<br><br>PFOA<br>86.5 ±177.2<br><br>PFOS<br>25.1 ± 16.7<br><br>PFNA<br>% (n) ng/mL<br>1.5 (0.77)<br><br><u>No diabetes</u><br><br>PFHxS<br>5.2 ± 10.4<br><br>PFOA<br>82.3 ± 227.2<br><br>PFOS<br>23.1 ± 15.4	Serum samples	PFHxS, PFOA, PFOS and PFNA levels were negatively associated with diabetes in age and sex adjusted analyses.	Adjusted OR (95% CI) for uncategorized Diabetes	PFHxS<br>**0.84** (0.78–0.90)<br><br>PFOA<br>**0.92** (0.88–0.97)<br><br>PFOS<br>0.93 (0.86–1.03)<br><br>PFNA<br>0.95 (0.85–1.06)	[11] Conway, B., K.E. Innes, and D. Long, Perfluoroalkyl substances and beta cell deficient diabetes. *J Diabetes Complications*, 2016. 30(6): p. 993-8.

		PFNA<br>% (n) ng/mL<br>1.6 (0.88)<br><br>Measured by high-performance liquid chromatography/tandem mass spectrometry with triple quadruple mass spectrometry.					

Table S5. PFAS & Insulin Resistance or Glucose Tolerance

Study Type/ Design<br><br>Study Sample Size (N)	Chemical Exposure(s)	Concentration Ranges and Detection Method(s)	Exposure Assessment	Summary of Results	Measures	Odds Ratio/Data	References
Cross-sectional analysis<br><br>571 adults	PFOA<br>PFOS<br>PFNA<br>PFUA	Median (IQR) (ng/mL)<br><br><u>Non-diabetes</u><br><br>PFOA<br>8.1 (6.0-10.6)<br><br>PFOS<br>3.1 (2.3-4.4)<br><br>PFNA<br>3.8 (2.7-5.1)<br><br>PFUA<br>6.5 (3.9-9.3)<br><br><u>Diabetes</u><br><br>PFOA<br>5.4 (2.5-7.6)<br><br>PFOS<br>5.2 (3.6-8.1)<br><br>PFNA<br>3.1 (1.2-4.8)<br><br>PFUA<br>4.3 (2.1-7.2)<br><br>Measured by UPLC coupled to triple quadrupole mass spectrometry.	Serum samples	PFOS exposure was positively associated with impaired glucose homeostasis and the prevalence of diabetes.<br><br>PFOA, PFNA, and PFUA showed a negative association with glucose intolerance and diabetes risk.	Adjusted OR (95% CI) for diabetes when comparing extreme quartiles	PFOS<br>3.37 (1.18–9.65)<br><br>PFOA<br>**0.16** (0.05–0.50)<br><br>PFNA<br>**0.31** (0.11–0.88)<br><br>PFUA<br>**0.23** (0.08–0.64)	[22] Su, T.C., et al., Serum perfluorinated chemicals, glucose homeostasis and the risk of diabetes in working-aged Taiwanese adults. *Environ Int*, 2016. 88: p. 15-22.
Prospective cohort study (NHANES)	PFOA<br>PFNA<br>PFOS<br>PFHxS	Mean ± SD (µg/L)<br><br>PFOA<br>4.6 ± 3.0	Serum sample	No association between PFOA, PFNA, PFOS, and PFHxS and HOMA-IR	Adjusted Log HOMA-IR beta coefficients (95% CI) for men and	PFOA<br>0.15 (-0.14, 0.44)<br><br>PFNA<br>0.18 (-0.06, 0.42)	[26] Nelson, J.W., E.E. Hatch, and T.F. Webster, Exposure to polyfluoroalkyl chemicals and

524 individuals		PFOS<br>25.3 ± 20.6<br><br>PFNA<br>1.3 ± 1.2<br><br>PFHxS<br>2.6 ± 2.7<br><br>Measured by solid-phase extraction coupled to isotope dilution/high-performance liquid chromatography/tandem mass spectrometry.			women age 20-80 for quartile 4	PFOS<br>0.12 (-0.17, 0.42)<br><br>PFHxS<br>0.05 (-0.18, 0.29)	cholesterol, body weight, and insulin resistance in the general U.S. population. *Environ Health Perspect*, 2010. 118(2): p. 197-202.
Cross-sectional analysis (NHANES)<br><br>474 adolescents and 969 adults	PFOA<br>PFOS<br>PFHS<br>PFNA	Mean ± SEM (ng/mL)<br><br><u>Adolescents</u><br><br>Log PFHS<br>0.95 ± 0.10<br><br>Log PFNA<br>-0.35 ± 0.07<br><br>Log PFOA<br>1.51 ± 0.05<br><br>Log PFOS<br>3.11 ± 0.05<br><br><u>Adults</u><br><br>Log PFHS<br>0.60 ±0.04<br><br>Log PFNA<br>-0.21 ± 0.07<br><br>Log PFOA<br>1.48 ± 0.04<br><br>Log PFOS<br>3.19 ± 0.04	Serum Samples	Serum levels of PFNA were positively associated with hyperglycemia while PFOA concentrations were associated with increased beta-cell function. PFOS levels were associated with both an increase in blood insulin, beta-cell function and the HOMA-IR.	OR (95% CI) for hyperglycemia<br><br>Model values (95% CI) of homeostatic model assessment of insulin resistance (HOMA-IR), β-cell function (HOMA-β), and blood insulin	<u>Hyperglycemia</u><br>PFNA<br>3.16 (1.39-7.16)<br><br><u>Beta-Cell function</u><br>PFOA<br>0.07 +/- 0.03<br><br>PFOS<br>0.15 +/- 0.05<br><br><u>Blood insulin</u><br>PFOS<br>0.14 +/- 0.05<br><br><u>HOMA-IR</u><br>PFOS<br>0.14 +/- 0.05	[27] Lin, C.Y., et al., Association among serum perfluoroalkyl chemicals, glucose homeostasis, and metabolic syndrome in adolescents and adults. *Diabetes Care*, 2009. 32(4): p. 702-7.

		Measured by high-performance liquid chromatography with negative-ion TurboIonSpray ionization tandem mass spectrometry.					
Cohort<br><br>40 overweight and obese Hispanic children (8-14 years)	PFOA<br>PFOS<br>PFHxS	Geometric mean (min-max) (ng/mL)<br><br>PFHxS<br>1.65 (0.47-12.81)<br><br>PFOS<br>12.22 (1.95-65.3)<br><br>PFOA<br>2.78 (1.88-5.37)<br><br>Measured by LC-HRMS with reverse phase chromatography and negative mode ESI.	Plasma samples	PFOA and PFHxS were associated with an increase in 2-hour glucose levels with each natural log increase in PFAS concentration.	2-hour glucose levels (95% CI)	PFOA<br>30.6 mg/dL (8.8-52.4)<br><br>PFHxS<br>10.2 mg/dL (2.7-17.7)	[28] Alderete, T.L., et al., Perfluoroalkyl substances, metabolomic profiling, and alterations in glucose homeostasis among overweight and obese Hispanic children: A proof-of-concept analysis. *Environ Int*, 2019. 126: p. 445-453.
Cross-sectional analysis (NHANES)<br><br>2,975 individuals	PFDA<br>PFOA<br>PFOS<br>PFHxS<br>MPAH<br>PFNA<br>PFUnDA	Median (25,75th percentiles) (µg/L)<br><br>PFDA<br>0.2 (0.1,0.4)<br><br>PFOA<br>2.8 (1.8,4.3)<br><br>PFOS<br>8.4 (4.8,14.0)<br><br>PFHxS<br>1.6 (0.9,2.8)<br><br>MPAH<br>0.2 (0.07,0.3)<br><br>PFNA<br>1.0 (0.7,1.5)<br><br>PFUnDA	Serum samples	PFUnDA was negatively associated with elevated glucose, which is a component of metabolic syndrome.	OR (95% CI) for elevated glucose when comparing quartile 3 and 1	PFUnDA<br>0.64 (0.42,0.98)	[29] Christensen, K.Y., M. Raymond, and J. Meiman, Perfluoroalkyl substances and metabolic syndrome. *Int J Hyg Environ Health*, 2019. 222(1): p. 147-153.

		0.1 (0.1,0.2)<br><br>Measured by solid phase extraction coupled to high performance liquid chromatography-turbo ion spray ionization-tandem mass spectrometry					
Cross-sectional study<br><br>311 young girls	PFOA<br>Me-PFOSA-AcOH<br>PFDeA<br>PFOS<br>PFHxS<br>PFNA	Median (min-max) (ng/mL)<br><br>Me-PFOSA-AcOH<br>0.80 (<LOD-9.30)<br><br>PFDeA<br>0.30 (<LOD-1.00)<br><br>PFHxS<br>5.20 (<LOD-185.00)<br><br>PFNA<br>1.40 (<LOD-6.80)<br><br>PFOA<br>7.30 (<LOD-55.90)<br><br>PFOS<br>13.60 (<LOD-96.00)<br><br>Measured by online solid-phase extraction high performance liquid chromatography–tandem mass spectrometry.	Blood samples	PFOA had an inverse association with insulin resistance, but it was not significant.	Linear Regression Beta coefficient (β) on HOMA-IR	PFOA<br>β= -0.1025<br><br>p=0.0864	[30] Fassler, C.S., et al., Complex relationships between perfluorooctanoate, body mass index, insulin resistance and serum lipids in young girls. *Environ Res*, 2019. 176: p. 108558.
Prospective cohort study<br><br>Adolescents (n = 201) and young adults (n = 202	PFOS<br>PFOA	Median (IQR) (ng/mL)<br><br><u>Childhood- Males</u><br><br>PFOS<br>44.5 (35.4-55.7)<br><br>PFOA	Plasma samples	Previous childhood exposure around age 9 to PFOS and PFOA was associated with a decreased β-cell function at 15 years of age.	Percent change (median) on β-cell function by 10ng/mL increase in exposure	PFOS<br>2% change in decreased β-cell function<br><br>PFOA	[31] Domazet, S.L., et al., Longitudinal Associations of Exposure to Perfluoroalkylated Substances in Childhood and Adolescence and

		9.7 (7.7-12.1)<br><br><u>Adolescence- Males</u><br><br>PFOS<br>22.3 (16.5-25.1)<br><br>PFOA<br>3.7 (2.7-4.4)<br><br><u>Adulthood-Males</u><br>PFOS<br>11.9 (9.2-15.2)<br><br>PFOA<br>3.1 (2.5-3.9)<br><br><u>Childhood- Females</u><br><br>PFOS<br>39.9 (34.3-49.3)<br><br>PFOA<br>9.0 (7.4-11.2)<br><br><u>Adolescence- Females</u><br><br>PFOS<br>20.8 (15.9-24.7)<br><br>PFOA<br>3.4 (2.8-4.5)<br><br><u>Adulthood-Females</u><br><br>PFOS<br>9.1 (7.0-10.8)<br><br>PFOA<br>2.7 (2.1-3.4)<br><br>Measured by on-line solid phase extraction followed by liquid chromatography and triple quadropole mass spectrometry (LC-MS/MS).				12% change in decreased β-cell function	Indicators of Adiposity and Glucose Metabolism 6 and 12 Years Later: The European Youth Heart Study. *Diabetes Care,* 2016. 39(10): p. 1745-51.

Cross-sectional study<br><br>2,700 individuals	PFOA<br>PFOS<br>PFHxS	Geometric mean (SE) (μg/L)<br><br>PFOS<br>8.40 (2.04)<br><br>PFOA<br>2.46 (1.83)<br><br>PFHxS<br>2.18 (2.63)<br><br>Measured by UPLC-MS/MS with an electrospray ion source in the negative mode.	Plasma samples	PFOA, PFOS, and PFHxS had no significant associations with plasma glucose, plasma insulin, or HOMA-IR.	Adjusted linear regression beta coefficients with (P-values) weighted model of data	<u>Plasma Insulin</u><br>PFOA<br>−0.071<br>(0.99)<br><br>PFOS<br>0.006<br>(0.18)<br><br>PFHxS<br>0.003<br>(0.07)<br><br><u>Plasma Glucose</u><br>PFOA<br>−0.04<br>(0.45)<br><br>PFOS<br>−0.0007<br>(0.28)<br><br>PFHxS<br>0.0002<br>(0.33)<br><br><u>HOMA-IR</u><br>PFOA<br>-0.1<br>(0.90)<br><br>PFOS<br>0.011<br>(0.11)<br><br>PFHxS<br>0.005<br>(0.07)	[32] Fisher, M., et al., Do perfluoroalkyl substances affect metabolic function and plasma lipids?--Analysis of the 2007-2009, Canadian Health Measures Survey (CHMS) Cycle 1. Environ Res, 2013. 121: p. 95-103.
Cross-sectional study<br><br>499 children	PFOS<br>PFOA	Median (range) (ng/mL)<br><br>PFOS<br>41.5 (6.2-132.5)<br><br>PFOA<br>9.3 (0.8-35.2)	Plasma samples	In overweight children both PFOA and PFOS were associated with increased insulin concentration, higher β-cell activity, and	Percent change (95% CI) for insulin concentration, B-cell activity, and insulin resistance.	<u>Insulin Concentration</u><br>PFOS<br>16.2% (5.2%-28.3%)<br><br>PFOA	[33] Timmermann, C.A., et al., Adiposity and glycemic control in children exposed to perfluorinated compounds. J Clin Endocrinol Metab,

		Measured by HPLC with LC-MS/MS.		elevated insulin resistance with an increase of 10 ng concentration of PFASs; no association was found in normal weight children.		71.6% (2.4%-187.5%)<br><br><u>β-cell activity</u><br>PFOS<br>12.0% (2.4%-22.4%)<br><br>PFOA<br>67.5% (5.5%-166.0%)<br><br><u>Insulin Resistance</u><br>PFOS<br>17.6% (5.8%-30.8%)<br><br>PFOA<br>73.9% (0.2%-202.0%)	2014. 99(4): p. E608-14.
cohort mother-child pairs (Project VIVA)<br><br>665 mother-child pairs	PFOA<br>PFOS<br>PFNA<br>PFHxS<br>PFDeA	Geometric mean (25th,75th percentile) (ng/mL)<br><br><u>Prenatal</u><br><br>PFOA<br>5.3 (3.9,7.6)<br><br>PFOS<br>24.4 (17.9,33.9)<br><br>PFNA<br>0.6 (0.5,0.9)<br><br>PFHxS<br>2.5 (1.6,3.8)<br><br><u>Mid-childhood</u><br><br>PFOA<br>4.2 (3.1,6.0)<br><br>PFOS<br>6.2 (4.2,9.7)	Maternal and childhood plasma	Higher PFOA levels in children were associated with a significantly lower insulin resistance level. This relationship was more pronounced in females than in males.	Model values (95% CI) of homeostatic model assessment of insulin resistance (HOMA-IR) per interquartile range (IQR)	<u>All Children</u><br>PFOA<br>-10.1% (-17.3, -2.3)<br><br><u>Females</u><br>PFOA<br>-15.6% (-25.4, -4.6)<br><br><u>Males</u><br>PFOA<br>-6.1% (-16.2, 5.2)	[34] Fleisch, A.F., et al., Early-Life Exposure to Perfluoroalkyl Substances and Childhood Metabolic Function. *Environ Health Perspect*, 2017. 125(3): p. 481-487.

		PFNA<br>1.7 (1.1,2.3)<br><br>PFHxS<br>2.2 (1.2,3.4)<br><br>Measured by on-line solid-phase extraction coupled to isotope dilution high performance liquid chromatography mass spectrometry.					
Cross-sectional analysis (NHANES)<br><br>1,871 adults	PFOA<br>PFOS<br>Linear PFOA<br>Branched PFOA<br>Linear PFOS<br>Branched PFOS	Geometric mean (SE) (ng/mL)<br><br><u>Total</u><br><br>Total PFOA<br>1.86 (1.02)<br><br>Linear PFOA<br>1.75 (1.02)<br><br>Branched PFOA<br>0.08 (1.01)<br><br>Total PFOS<br>5.28 (1.02)<br><br>Linear PFOS<br>3.70 (1.02)<br><br>Branched PFOS<br>1.39 (1.02)<br><br><u>HOMA-IR (≤ 2.36)</u><br><br>Total PFOA<br>1.87 (1.03)<br><br>Linear PFOA<br>1.75 (1.04)<br><br>Branched PFOA<br>0.08 (1.02)	Serum samples	Both PFOA (total and linear) and PFOS (branched) were positively correlated with enhanced β-cell function.	Adjusted log-total OR (95% CI) for glucose<br><br>Linear regression coefficients with standard error for β-cell function (HOMA-β) with (p value)	PFOA<br>**0.79**<br>(0.63–.994)<br><br>Log-Total PFOA 0.11 ± 0.04 for model 1 0.12 ± 0.05 for model 2 ($p < 0.05$)<br><br>Log- Linear PFOA 0.10 ± 0.04 for model 1 and 0.11 ± 0.04 for model 2 ($p < 0.05$)<br><br>log-branched PFOS<br>0.09 ± 0.03 for model 1 and 0.10 ± 0.04 for model 2 ($p < 0.05$)	[35] Liu, H.S., et al., Association among total serum isomers of perfluorinated chemicals, glucose homeostasis, lipid profiles, serum protein and metabolic syndrome in adults: NHANES, 2013-2014. *Environ Pollut*, 2018. 232: p. 73-79.

		Total PFOS<br>5.34 (1.05)<br><br>Linear PFOS<br>3.82 (1.05)<br><br>Branched PFOS<br>1.34 (1.05)<br><br>Measured by on-line solid phase extraction coupled to isotope-dilution high-performance liquid chromatography tandem mass spectrometry.					
Double-blind, randomized, placebo-controlled crossover trial<br><br>141 elderly subjects	PFBS<br>PFHxS<br>PFOS<br>PFDS<br>PFBA<br>PFPeA<br>PFHxA<br>PFHpA<br>PFOA<br>PFNA<br>PFDA<br>PFUnDA<br>PFDoDA<br>PFTrDA<br>PFTeDA	Mean ± SD (ng/mL)<br><br>PFBS<br>0.41 ± 0.13<br><br>PFHxS<br>0.23 ± 0.17<br><br>PFOS<br>10.04 ± 4.12<br><br>PFDS<br>0.25 ± 0.06<br><br>PFBA<br>0.86 ± 0.11<br><br>PFHpA<br>0.44 ± 0.31<br><br>PFOA<br>4.61 ± 1.86<br><br>PFNA<br>1.97 ± 0.94<br><br>PFDA<br>1.02 ± 0.41<br><br>PFUnDA	Serum Samples	PFOS and PFDoDA exposure was positively associated with insulin resistance. This article showed that a vitamin C supplement could protect against these effects.	HOMA-IR (95% CI) model values by IQR at the baseline and after placebo treatment	PFOS<br>0.60 (−0.002, 1.19)<br><br>PFDoDA<br>0.83 (0.23, 1.44)	[36] Kim, J.H., et al., The modifying effect of vitamin C on the association between perfluorinated compounds and insulin resistance in the Korean elderly: a double-blind, randomized, placebo-controlled crossover trial. *Eur J Nutr*, 2016. 55(3): p. 1011-20.

		2.19 ± 0.96<br><br>PFDoDA<br>0.25 ± 0.09<br><br>PFTrDA<br>0.50 ± 0.21<br><br>PFTeDA<br>0.25 ± 0.11<br><br>Measured by HPLC coupled with triple–quadruple mass spectrometry.					
Case-control<br><br>308 participants, 123 in the WTCHR group and 185 in the comparison group	PFOA<br>PFHxS<br>PFOS<br>PFNA<br>PFDA<br>PFUnDA	Median (IQR) (ng/mL)<br><br>World Trade Center cohort<br><br>PFHxS<br>0.67 (0.69)<br><br>PFOS<br>3.72 (2.82)<br><br>PFOA<br>1.81 (0.90)<br><br>PFNA<br>0.61 (0.36)<br><br>PFDA<br>0.14 (0.12)<br><br>PFUnDA<br>0.12 (0.21)<br><br>Comparison group<br><br>PFHxS<br>0.53 (0.47)<br><br>PFOS<br>2.78 (2.18)<br><br>PFOA	Serum samples	PFHxS levels were associated with decreased insulin resistance.	Percent change of HOMA-IR (95% CI)<br><br>logistic regression beta coefficient (95% CI)	HOMA-IR<br>PFHxS<br>-8.6% (-16.1,-0.3)<br><br>Beta-Coefficient<br>PFHxS<br>−0.090 (−0.176, −0.003)	[37] Koshy, T.T., et al., Serum perfluoroalkyl substances and cardiometabolic consequences in adolescents exposed to the World Trade Center disaster and a matched comparison group. *Environ Int*, 2017. 109: p. 128-135.

		1.39 (0.75)<br><br>PFNA<br>0.49 (0.33)<br><br>PFDA<br>0.11 (0.15)<br><br>PFUnDA<br>0.04 (0.16)<br><br>Measured by solid phase extraction and HPLC with electrospray tandem mass spectrometry.					
Pilot cross sectional study<br><br>123 individuals	PFOA<br>PFOS<br>PFNA<br>PFHxS	Geometric mean (range) ng/mL<br><br>PFOS<br>8.91 (2.36,33.67)<br><br>PFOA<br>2.87 (1.03,8.02)<br><br>PFHxS<br>0.77 (0.25, 2.40)<br><br>PFNA<br>1.29 (0.48,3.46)<br><br>Measured by online solid-phase extraction coupled to reversed-phase high-performance liquid chromatography-tandem mass spectrometry (HPLC/MS/MS).	Plasma samples	PFOA, PFOS, PFNA, and PFHxS had no statistically significant associations with fasting plasma glucose, fasting insulin, and HOMA-IR.	Model values (95% CI) of higher β-cell function (HOMA-β) per lnPFOS	PFOS<br>17.94 (−1.55, 37.44)	[38] Chen, A., et al., Association of perfluoroalkyl substances exposure with cardiometabolic traits in an island population of the eastern Adriatic coast of Croatia. *Sci Total Environ*, 2019. 683: p. 29-36.
Cross-sectional analysis (Diabetes Prevention Program)	Branched and linear isomers of PFOS and PFOA;<br>PFOA<br>n-PFOS<br>Sm-PFOS	Geometric mean (IQR) (ng/mL)<br><br>Diabetes Prevention Program	Plasma samples	PFOA and PFOS concentration was associated with small differences in markers of insulin	Model values (95% CI) of homeostatic model assessment of insulin	PFOS<br>HOMA-IR<br>0.39 (0.13-0.66)<br><br>HOMA-β<br>9.62 (1.55-17.70)	[41] Cardenas, A., et al., Plasma Concentrations of Per- and Polyfluoroalkyl Substances at Baseline

957 adults	Sm2-PFOS<br>PFOA<br>n-PFOA<br>Sb-PFOA<br>PFHxS<br>Et-PFOSA-AcOH<br>Me-PFOSA-AcOH<br>PFNA	PFOA<br>26.38 (22.80)<br><br>n-PFOS<br>18.42 (16.90)<br><br>Sm-PFOS<br>7.32 (6.50)<br><br>Sm2-PFOS<br>0.13 (0.23)<br><br>PFOA<br>4.82 (3.20)<br><br>n-PFOA<br>4.29 (2.90)<br><br>Sb-PFOA<br>0.44 (0.50)<br><br>PFHxS<br>2.41 (2.40)<br><br>Et-PFOSA-AcOH<br>1.13 (1.50)<br><br>Me-PFOSA-AcOH<br>0.94 (1.10)<br><br>PFNA<br>0.53 (0.40)<br><br>Measured by online solid-phase extraction–high-performance liquid chromatography coupled to isotope dilution–tandem mass spectrometry		resistance and β-cell function.	resistance (HOMA-IR), higher β-cell function (HOMA-β), and higher fasting proinsulin (FP)	FP<br>1.37 pM (0.50-2.25)<br><br>PFOA<br>HOMA-IR<br>0.64 (0.34-0.94)<br><br>HOMA-β<br>15.93 (6.78-25.08)<br><br>FP<br>1.71 pM (0.72-2.71)	and Associations with Glycemic Indicators and Diabetes Incidence among High-Risk Adults in the Diabetes Prevention Program Trial. *Environ Health Perspect*, 2017. 125(10): p. 107001.

REFERENCES

1. Wang, Y.; Zhang, L.; Teng, Y.; Zhang, J.; Yang, L.; Li, J.; Lai, J.; Zhao, Y.; Wu, Y. Association of serum levels of perfluoroalkyl substances with gestational diabetes mellitus and postpartum blood glucose. *J. Environ. Sci.* **2018**, *69*, 5–11.
2. Zhang, C.; Sundaram, R.; Maisog, J.; Calafat, A.M.; Barr, D.B.; Buck Louis, G.M. A prospective study of prepregnancy serum concentrations of perfluorochemicals and the risk of gestational diabetes. *Fertil. Steril.* **2015**, *103*, 184–189.
3. Shapiro, G.D.; Dodds, L.; Arbuckle, T.E.; Ashley-Martin, J.; Ettinger, A.S.; Fisher, M.; Taback, S.; Bouchard, M.F.; Monnier, P.; Dallaire, R.; et al. Exposure to organophosphorus and organochlorine pesticides, perfluoroalkyl substances, and polychlorinated biphenyls in pregnancy and the association with impaired glucose tolerance and gestational diabetes mellitus: The MIREC Study. *Environ. Res.* **2016**, *147*, 71–81.
4. Preston, E.V.; Rifas-Shiman, S.L.; Hivert, M.F.; Zota, A.R.; Sagiv, S.K.; Calafat, A.M.; Oken, E.; James-Todd, T. Associations of Per- and Polyfluoroalkyl Substances (PFAS) With Glucose Tolerance During Pregnancy in Project Viva. *J. Clin. Endocrinol. Metab.* **2020**, *105*, e2864–e2876
5. Matilla-Santander, N.; Valvi, D.; Lopez-Espinosa, M.J.; Manzano-Salgado, C.B.; Ballester, F.; Ibarluzea, J.; Santa-Marina, L.; Schettgen, T.; Guxens, M.; Sunyer, J.; et al. Exposure to Perfluoroalkyl Substances and Metabolic Outcomes in Pregnant Women: Evidence from the Spanish INMA Birth Cohorts. *Environ. Health Perspect.* **2017**, *125*, 117004.
6. Wang, H.; Yang, J.; Du, H.; Xu, L.; Liu, S.; Yi, J.; Qian, X.; Chen, Y.; Jiang, Q.; He, G. Perfluoroalkyl substances, glucose homeostasis, and gestational diabetes mellitus in Chinese pregnant women: A repeat measurement-based prospective study. *Environ. Int.* **2018**, *114*, 12–20.
7. Rahman, M.L.; Zhang, C.; Smarr, M.M.; Lee, S.; Honda, M.; Kannan, K.; Tekola-Ayele, F.; Buck Louis, G.M. Persistent organic pollutants and gestational diabetes: A multi-center prospective cohort study of healthy US women. *Environ. Int.* **2019**, *124*, 249–258.
8. Liu, X.; Zhang, L.; Chen, L.; Li, J.; Wang, Y.; Wang, J.; Meng, G.; Chi, M.; Zhao, Y.; Chen, H.; et al. Structure-based investigation on the association between perfluoroalkyl acids exposure and both gestational diabetes mellitus and glucose homeostasis in pregnant women. *Environ. Int.* **2019**, *127*, 85–93.
9. Jensen, R.C.; Glintborg, D.; Timmermann, C.A.G.; Nielsen, F.; Kyhl, H.B.; Andersen, H.R.; Grandjean, P.; Jensen, T.K.; Andersen, M. Perfluoroalkyl substances and glycemic status in pregnant Danish women: The Odense Child Cohort. *Environ. Int.* **2018**, *116*, 101–107.
10. Xu, H.; Zhou, Q.; Zhang, J.; Chen, X.; Zhao, H.; Lu, H.; Ma, B.; Wang, Z.; Wu, C.; Ying, C.; et al. Exposure to elevated per- and polyfluoroalkyl substances in early pregnancy is related to increased risk of gestational diabetes mellitus: A nested case-control study in Shanghai, China. *Environ. Int.* **2020**, *143*, 105952.
11. Conway, B.; Innes, K.E.; Long, D. Perfluoroalkyl substances and beta cell deficient diabetes. *J. Diabetes Complicat.* **2016**, *30*, 993–998.
12. Predieri, B.; Iughetti, L.; Guerranti, C.; Bruzzi, P.; Perra, G.; Focardi, S.E. High Levels of Perfluorooctane Sulfonate in Children at the Onset of Diabetes. *Int. J. Endocrinol.* **2015**, *2015*, 234358.
13. Steenland, K.; Zhao, L.; Winquist, A.; Parks, C. Ulcerative colitis and perfluorooctanoic acid (PFOA) in a highly exposed population of community residents and workers in the mid-Ohio valley. *Environ. Health Perspect.* **2013**, *121*, 900–905.
14. Sun, Q.; Zong, G.; Valvi, D.; Nielsen, F.; Coull, B.; Grandjean, P. Plasma Concentrations of Perfluoroalkyl Substances and Risk of Type 2 Diabetes: A Prospective Investigation among U.S. Women. *Environ. Health Perspect.* **2018**, *126*, 037001.
15. Donat-Vargas, C.; Bergdahl, I.A.; Tornevi, A.; Wennberg, M.; Sommar, J.; Kiviranta, H.; Koponen, J.; Rolandsson, O.; Åkesson, A. Perfluoroalkyl substances and risk of type II diabetes: A prospective nested case-control study. *Environ. Int.* **2019**, *123*, 390–398.
16. Karnes, C.; Winquist, A.; Steenland, K. Incidence of type II diabetes in a cohort with substantial exposure to perfluorooctanoic acid. *Environ. Res.* **2014**, *128*, 78–83.
17. Mancini, F.R.; Rajaobelina, K.; Praud, D.; Dow, C.; Antignac, J.P.; Kvaskoff, M.; Severi, G.; Bonnet, F.; Boutron-Ruault, M.C.; Fagherazzi, G. Nonlinear associations between dietary exposures to perfluorooctanoic acid (PFOA) or perfluorooctane sulfonate (PFOS) and type 2 diabetes risk in women: Findings from the E3N cohort study. *Int. J. Hyg. Environ. Health* **2018**, *221*, 1054–1060.
18. MacNeil, J.; Steenland, N.K.; Shankar, A.; Ducatman, A. A cross-sectional analysis of type II diabetes in a community with exposure to perfluorooctanoic acid (PFOA). *Environ Res* **2009**, *109*, 997–1003.

19. Cardenas, A.; Hivert, M.F.; Gold, D.R.; Hauser, R.; Kleinman, K.P.; Lin, P.D.; Fleisch, A.F.; Calafat, A.M.; Ye, X.; Webster, T.F.; et al. Associations of Perfluoroalkyl and Polyfluoroalkyl Substances With Incident Diabetes and Microvascular Disease. *Diabetes Care* **2019**, *42*, 1824–1832.
20. He, X.; Liu, Y.; Xu, B.; Gu, L.; Tang, W. PFOA is associated with diabetes and metabolic alteration in US men: National Health and Nutrition Examination Survey 2003–2012, *Sci. Total Environ.* **2018**, *625*, 566–574.
21. Lind, L.; Zethelius, B.; Salihovic, S.; van Bavel, B.; Lind, P.M. Circulating levels of perfluoroalkyl substances and prevalent diabetes in the elderly. *Diabetologia* **2014**, *57*, 473–479.
22. Su, T.C.; Kuo, C.C.; Hwang, J.J.; Lien, G.W.; Chen, M.F.; Chen, P.C. Serum perfluorinated chemicals, glucose homeostasis and the risk of diabetes in working-aged Taiwanese adults. *Environ. Int.* **2016**, *88*, 15–22.
23. Seo, S.-H.; Son, M.-H.; Choi, S.-D.; Lee, D.-H.; Chang, Y.-S. Influence of exposure to perfluoroalkyl substances (PFASs) on the Korean general population: 10-year trend and health effects. *Environ. Int.* **2018**, *113*, 149–161.
24. Lundin, J.I.; Alexander, B.H.; Olsen, G.W.; Church, T.R. Ammonium perfluorooctanoate production and occupational mortality. *Epidemiology* **2009**, *20*, 921–928.
25. Christensen, K.Y.; Raymond, M.; Thompson, B.A.; Anderson, H.A. Perfluoroalkyl substances in older male anglers in Wisconsin. *Environ. Int.* **2016**, *91*, 312–318.
26. Nelson, J.W.; Hatch, E.E.; Webster, T.F. Exposure to polyfluoroalkyl chemicals and cholesterol, body weight, and insulin resistance in the general U.S. population. *Environ. Health Perspect.* **2010**, *118*, 197–202.
27. Lin, C.Y.; Chen, P.C.; Lin, Y.C.; Lin, L.Y. Association among serum perfluoroalkyl chemicals, glucose homeostasis, and metabolic syndrome in adolescents and adults. *Diabetes Care* **2009**, *32*, 702–707.
28. Alderete, T.L.; Jin, R.; Walker, D.I.; Valvi, D.; Chen, Z.; Jones, D.P.; Peng, C.; Gilliland, F.D.; Berhane, K.; Conti, D.V.; et al. Perfluoroalkyl substances, metabolomic profiling, and alterations in glucose homeostasis among overweight and obese Hispanic children: A proof-of-concept analysis. *Environ. Int.* **2019**, *126*, 445–453.
29. Christensen, K.Y.; Raymond, M.; Meiman, J. Perfluoroalkyl substances and metabolic syndrome. *Int. J. Hyg. Environ. Health* **2019**, *222*, 147–153.
30. Fassler, C.S.; Pinney, S.E.; Xie, C.; Biro, F.M.; Pinney, S.M. Complex relationships between perfluorooctanoate, body mass index, insulin resistance and serum lipids in young girls. *Environ. Res.* **2019**, *176*, 108558.
31. Domazet, S.L.; Grøntved, A.; Timmermann, A.G.; Nielsen, F.; Jensen, T.K. Longitudinal Associations of Exposure to Perfluoroalkylated Substances in Childhood and Adolescence and Indicators of Adiposity and Glucose Metabolism 6 and 12 Years Later: The European Youth Heart Study. *Diabetes Care* **2016**, *39*, 1745–1751.
32. Fisher, M.; Arbuckle, T.E.; Wade, M.; Haines, D.A. Do perfluoroalkyl substances affect metabolic function and plasma lipids?—Analysis of the 2007–2009, Canadian Health Measures Survey (CHMS) Cycle 1. *Environ. Res.* **2013**, *121*, 95–103.
33. Timmermann, C.A.; Rossing, L.I.; Grøntved, A.; Ried-Larsen, M.; Dalgård, C.; Andersen, L.B.; Grandjean, P.; Nielsen, F.; Svendsen, K.D.; Scheike, T.; et al. Adiposity and glycemic control in children exposed to perfluorinated compounds. *J. Clin. Endocrinol. Metab.* **2014**, *99*, E608–E614.
34. Fleisch, A.F.; Rifas-Shiman, S.L.; Mora, A.M.; Calafat, A.M.; Ye, X.; Luttmann-Gibson, H.; Gillman, M.W.; Oken, E.; Sagiv, S.K. Early-Life Exposure to Perfluoroalkyl Substances and Childhood Metabolic Function. *Environ. Health Perspect.* **2017**, *125*, 481–487.
35. Liu, H.S.; Wen, L.L.; Chu, P.L.; Lin, C.Y. Association among total serum isomers of perfluorinated chemicals, glucose homeostasis, lipid profiles, serum protein and metabolic syndrome in adults: NHANES, 2013–2014. *Environ. Pollut.* **2018**, *232*, 73–79.
36. Kim, J.H.; Park, H.Y.; Jeon, J.D.; Kho, Y.; Kim, S.K.; Park, M.S.; Hong, Y.C. The modifying effect of vitamin C on the association between perfluorinated compounds and insulin resistance in the Korean elderly: A double-blind, randomized, placebo-controlled crossover trial. *Eur. J. Nutr.* **2016**, *55*, 1011–1020.
37. Koshy, T.T.; Attina, T.M.; Ghassabian, A.; Gilbert, J.; Burdine, L.K.; Marmor, M.; Honda, M.; Chu, D.B.; Han, X.; Shao, Y.; et al. Serum perfluoroalkyl substances and cardiometabolic consequences in adolescents exposed to the World Trade Center disaster and a matched comparison group. *Environ. Int.* **2017**, *109*, 128–135.
38. Chen, A.; Jandarov, R.; Zhou, L.; Calafat, A.M.; Zhang, G.; Urbina, E.M.; Sarac, J.; Augustin, D.H.; Caric, T.; Bockor, L.; et al. Association of perfluoroalkyl substances exposure with cardiometabolic traits in an island population of the eastern Adriatic coast of Croatia. *Sci. Total Environ.* **2019**, *683*, 29–36.
39. Conway, B.N.; Badders, A.N.; Costacou, T.; Arthur, J.M.; Innes, K.E. Perfluoroalkyl substances and kidney function in chronic kidney disease, anemia, and diabetes. *Diabetes Metab. Syndr. Obes.* **2018**, *11*, 707–716.
40. Girardi, P.; Merler, E. A mortality study on male subjects exposed to polyfluoroalkyl acids with high internal dose of perfluorooctanoic acid. *Environ. Res.* **2019**, *179*, 108743
41. Cardenas, A.; Gold, D.R.; Hauser, R.; Kleinman, K.P.; Hivert, M.F.; Calafat, A.M.; Ye, X.; Webster, T.F.; Horton, E.S.; Oken, E. Plasma Concentrations of Per- and Polyfluoroalkyl Substances at Baseline and Associations with

Glycemic Indicators and Diabetes Incidence among High-Risk Adults in the Diabetes Prevention Program Trial. *Environ. Health Perspect.* **2017**, *125*, 107001.
